# Multi-objective optimization and algorithmic evaluation for EMS in a HRES integrating PV, wind, and backup storage

**DOI:** 10.1038/s41598-024-84227-0

**Published:** 2025-01-07

**Authors:** Ahmed A. Shaier, Mahmoud M. Elymany, Mohamed A. Enany, Nadia A. Elsonbaty

**Affiliations:** Electrical Power and Machines Department, Faculty of Engineering, Zagazig University, Zagazig, 44519 Egypt

**Keywords:** Hybrid backup system, Smart power flow management, Honey badger algorithm (HBA), Supercapacitors, Hybrid renewable energy system, Solar energy, Wind energy, Electrical and electronic engineering

## Abstract

This manuscript focuses on optimizing a Hybrid Renewable Energy System (HRES) that integrates photovoltaic (PV) panels, wind turbines (WT), and various energy storage systems (ESS), including batteries, supercapacitors (SCs), and hydrogen storage. The system uses a multi-objective optimization strategy to balance power management, aiming to minimize costs and reduce the likelihood of loss of power supply probability (LPSP). Seven different algorithms are assessed to identify the most efficient one for achieving these objectives, with the goal of selecting the algorithm that best balances cost efficiency and system performance. The system is assessed across three operational scenarios: (1) when energy supply meets demand with help from backup systems, (2) when demand exceeds supply and energy storage systems are depleted, and (3) when energy generation surpasses demand and storage systems are full. The HBA-based optimization effectively manages energy flow and storage, ensuring grid stability and minimizing overcharging risks. This system offers a reliable and sustainable power supply for isolated microgrids, effectively managing energy production, storage, and distribution. The research sets a new benchmark for future studies in decentralized energy systems, particularly in balancing technical efficiency and economic feasibility.

## Introduction

Microgrids have become a practical solution to tackle critical energy issues, such as supply shortages and mismatches between generation and consumption. These localized energy systems provide several advantages, including the ability to cut greenhouse gas emissions, strengthen energy security, and create a more dependable and resilient energy framework. Recent developments in microgrid technology have concentrated on enhancing their efficiency and performance^1^. A significant area of advancement is the integration of renewable energy sources (RESs) into microgrids. These systems frequently include solar panels, wind turbines, and biomass to create a more diverse energy mix. Additionally, state-of-the-art energy storage solutions, like advanced batteries, facilitate the smooth integration of variable renewable sources by storing surplus energy for use when generation is low^2,3^. The adoption of smart grid technologies has been vital in enhancing the efficiency of microgrid operations. These technologies utilize advanced communication and control systems to effectively manage the generation, distribution, and consumption of energy^4^. Smart grids allow for real-time monitoring and dynamic adjustment of supply and demand, improving overall efficiency. Predictive analytics and forecasting tools have become essential components in the management of microgrids^5^. These tools enable operators to predict energy demand trends and strategically manage the allocation of resources. This leads to a more efficient microgrid, ensuring that resources are deployed effectively to meet future energy needs. Energy Management Systems (EMS) are key elements that enhance the performance of microgrids by optimizing their operations^6,7^. These systems leverage real-time data and analytics to make informed decisions regarding energy production, storage, and distribution. By effectively balancing the load and managing resources, Energy Management Systems (EMS) enhance the overall stability and reliability of microgrids. Additionally, decentralized control systems are increasingly replacing or supplementing traditional centralized control approaches. These systems provide greater flexibility and adaptability in managing microgrid components, improving resilience and responsiveness to shifts in the energy environment. Together, these advancements make microgrids more efficient, resilient, and capable of addressing challenges related to energy shortages and sustainability. As technology continues to progress, microgrids are set to play an increasingly crucial role in the future of energy systems^8–10^.

The researchers highlight the importance of energy management systems (EMS) in regulating the balance between energy supply and demand within microgrids. This is especially crucial in renewable energy systems, where power generation from sources like solar panels and wind turbines can be variable and unpredictable^11^. EMS optimize the use of available energy resources, ensuring a reliable and stable power supply. By providing advanced analytics and optimization algorithms, EMS supports informed decision-making across various operations. These capabilities enable predictive maintenance of equipment, efficient scheduling of energy resources, and the identification of opportunities for demand response and energy trading. Utilizing data analytics and machine learning techniques, EMS can continually improve its performance, adapting to changing energy demands and market conditions. Furthermore, EMS is essential for managing distributed energy resources within microgrids, coordinating their operation for maximum efficiency and reliability^12,13^. Distributed Energy Resources (DERs) encompass a range of renewable energy sources, energy storage systems, and even electric vehicles. The Energy Management System (EMS) coordinates the operation of these resources, ensuring that energy is produced, stored, and consumed as efficiently as possible. EMS also oversees power dispatch within microgrids, determining how much energy should be generated by each source, how much should be stored, and how much should be used. By factoring in variables like energy prices, system constraints, and user preferences, the EMS makes informed decisions to optimize microgrid performance. Additionally, EMS facilitates the integration of renewable energy sources (RESs) and energy storage, ensuring smooth incorporation of intermittent renewable generation into the microgrid while maintaining grid stability. With advanced control strategies, EMS maximizes renewable energy usage, stores excess energy when generation exceeds demand, and dispatches stored energy during periods of high demand or low renewable output. Another crucial function of EMS is supporting islanded operation. In case of a grid outage or as part of a resilience strategy, the microgrid can disconnect from the main grid and operate independently. The EMS manages this transition, ensuring stability in frequency and voltage, while reconfiguring the system to sustain essential loads. Furthermore, the EMS enables demand-side management and consumer engagement within the microgrid. It supports demand response programs, time-of-use pricing, and interactive communication with consumers to encourage energy conservation and flexible load management. By empowering users to actively participate in energy management, the EMS helps create a more sustainable and responsive energy ecosystem^14^.

The importance of diverse energy storage systems such as batteries, supercapacitors, and fuel cells lie in their complementary strengths, which make them essential for supporting modern energy infrastructure, particularly as we transition to renewable energy sources. The authors in^15^ review the capabilities of energy storage systems (ESSs) with a focus on their current applications on non-interconnected European islands. This review emphasizes the essential role of storage systems in supporting the increased integration of renewable energy sources (RES) into the energy mix. It examines various electrochemical, mechanical, and thermal storage technologies, comparing them across criteria such as energy density, efficiency, nominal power, environmental impact, and lifespan, while highlighting the extensive capabilities of batteries and pumped hydro storage systems, especially in large-scale networks. Additionally, it explores ancillary services like frequency and voltage regulation, underlining the value of controllable storage systems as sustainable alternatives to fossil fuel generators in modern power systems. Real-world installations of ESSs on non-interconnected European islands are also analyzed, with a particular emphasis on nominal power and voltage. The study details notable examples, including the pumped hydro storage system on Ikaria, Greece, and a complex storage system on the Portuguese island of Terceira, which integrates a battery, vehicle-to-grid (V2G), and flywheel technology. The aim is to provide an overview of current storage installations and facilitate the adoption of ESSs in more power systems in the future. Batteries, such as lithium-ion, are known for their high energy density, which allows them to store significant amounts of energy for longer durations. This makes them suitable for applications like electric vehicles and grid energy storage where sustained power is critical. Fuel cells also have high energy densities, making them ideal for applications requiring extended operation without frequent recharging^16^. Supercapacitors, on the other hand, excel in delivering high power density with rapid charge and discharge cycles. They are best suited for applications where quick bursts of energy are needed, such as in frequency regulation or for critical pulse loads in transportation systems and microgrids. However, they are limited by lower energy densities compared to batteries^16,17^. By integrating multiple storage systems into hybrid energy setups, we can leverage the high energy density of batteries and fuel cells with the fast response times of supercapacitors. This combination enhances overall system resilience and flexibility, addressing diverse energy needs from renewable grid support to high-demand industrial applications​^16^. Ultimately, a diverse array of energy storage solutions is critical for optimizing the balance between power availability, efficiency, and reliability across various modern energy applications.

Genetic algorithms, metaheuristic methods, fuzzy logic, and Artificial Neural Networks (ANN) are identified as promising approaches for optimizing the design and operation of HRESs within microgrids. These techniques can assist in determining the most effective combination of energy sources, storage solutions, and power dispatch strategies to enhance both efficiency and reliability^18,19^. Several studies have shown the effectiveness of GAs in optimizing the design and operation of HRESs. For instance, research^20^ utilized GAs to optimize the sizing and management of a hybrid green energy system that included solar panels, wind turbines, and batteries. Another study applied GAs to minimize the overall operating costs of a PV-wind-diesel-battery system through model predictive control^21^. Beyond optimizing system design and operation, GAs have also been employed to improve other aspects of HRESs, such as the strategic placement of energy storage devices. In study^22^, Genetic Algorithms (GAs) were used to optimize the topology and sizing of distributed energy storage systems in domestic photovoltaic (PV) systems connected to low-voltage networks. Particle Swarm Optimization (PSO) has also been widely applied to various tasks, such as optimal sizing, scheduling, and economic dispatch of systems combining solar, wind, fuel cells, and batteries. PSO has proven effective in real-time controller tuning to regulate power in grid-connected hybrid systems^23^. In research^24^, PSO was employed to optimize the energy management system for a stand-alone Hybrid Renewable Energy System (HRES). The PSO method adjusted the proportional-integral (PI) controller gains based on the power generated by key resources like PV and wind to calculate battery power generation. Additionally, in study^25^, PSO was used to optimize the design of hybrid PV/battery and off-grid PV/hydrogen systems for remote areas. The analysis focused on the system’s reliability, the number of simulations runs, and interest rates. Results showed that the PV/battery system had a total configured capacity of solar panels 44.8% smaller than the PV/hydrogen system and observed a 2.2% reduction in total annual costs as the number of runs increased for the PV/hydrogen system.

Fuzzy Logic is widely applied in Hybrid Renewable Energy Systems (HRESs) for tasks such as regulating inverters to control grid-injected current and managing real/reactive power. By developing a robust Fuzzy Logic Controller (FLC), oscillations can be reduced, and the controller’s performance can be improved, even in the face of external disturbances or fluctuations in load demand or power generation. Optimal system operation is achieved by efficiently managing the battery’s State of Charge (SOC), which helps to reduce peak current demand and extend the overall lifespan of the system^26–28^. Surplus power is utilized to lower operational costs and enhance the reliability of hybrid systems, with the help of an iterative Pareto-fuzzy algorithm^29^. The fuzzy multi-objective design of hybrid green power systems, which takes into account factors like cost, reliability, and environmental emissions, has also been investigated in^30^. Additionally, controllers designed to ensure smooth power flow transitions in real-time environments have been experimentally validated in studies^31^, and^32^. In study^1^, a highly efficient Hybrid Renewable Energy System (HRES) is proposed, combining photovoltaic and wind energy sources with battery, hydrogen, and supercapacitor storage. The system uses a Zebra Optimization Algorithm (ZOA)-ANFIS-based Maximum Power Point Tracking (MPPT) and an Artificial Gorilla Troops Optimizer (AGTO)-ANFIS-based MPPT to optimize energy flow. A MATLAB/Simulink-based power flow management model demonstrates effective performance under various scenarios. A comparative analysis shows that the ZOA technique outperforms others, reducing computation time by 26.17% for the photovoltaic system and 35.5% for the wind energy conversion system. In^33^, an Artificial Gorilla Troop Optimizer was introduced for managing energy consumption in DC-AC hybrid distribution networks using artificial neural networks (ANN). The proposed energy management system takes into account distributed generation, load demand, and battery charge levels. The ANN was trained on the characteristics of the energy storage system, achieving a low error rate. The system employs the gorilla-inspired optimizer to adjust network weights and biases based on power parameters. Small-scale hybrid DC/AC microgrids were developed and tested, showing a remarkable 99.55% efficiency compared to other systems in the literature.

Recent optimization algorithms have been increasingly applied to enhance the performance and reduce the overall cost of Hybrid Renewable Energy Systems (HRES). This study focuses on implementing an innovative intelligent energy management system, with the following main contributions:

- Developing an advanced HRES that integrates PV panels and WTs as the primary power sources, with batteries, fuel cells, and SCs serving as three backup storage options.
- Investigating the proposed EMS designed to manage the system under three distinct scenarios.
- Comparing the HBA-based EMS with other optimization methods like GOA, GTO, MRFO, SSOA, SEA, and POA to to determine the most effective method.
- Analyzing various EMS performance factors, including LPSP, system efficiency, and convergence time, to determine the best optimization algorithm for the system.
- The proposed EMS demonstrates outstanding overflow management capabilities, essential for preserving the health and longevity of the ESS. This feature helps prevent premature wear and tear, ensuring long-term reliability and optimal operation, which prolongs the ESS’s lifespan.

## Design of the proposed HRES system

**Fig. 1:**
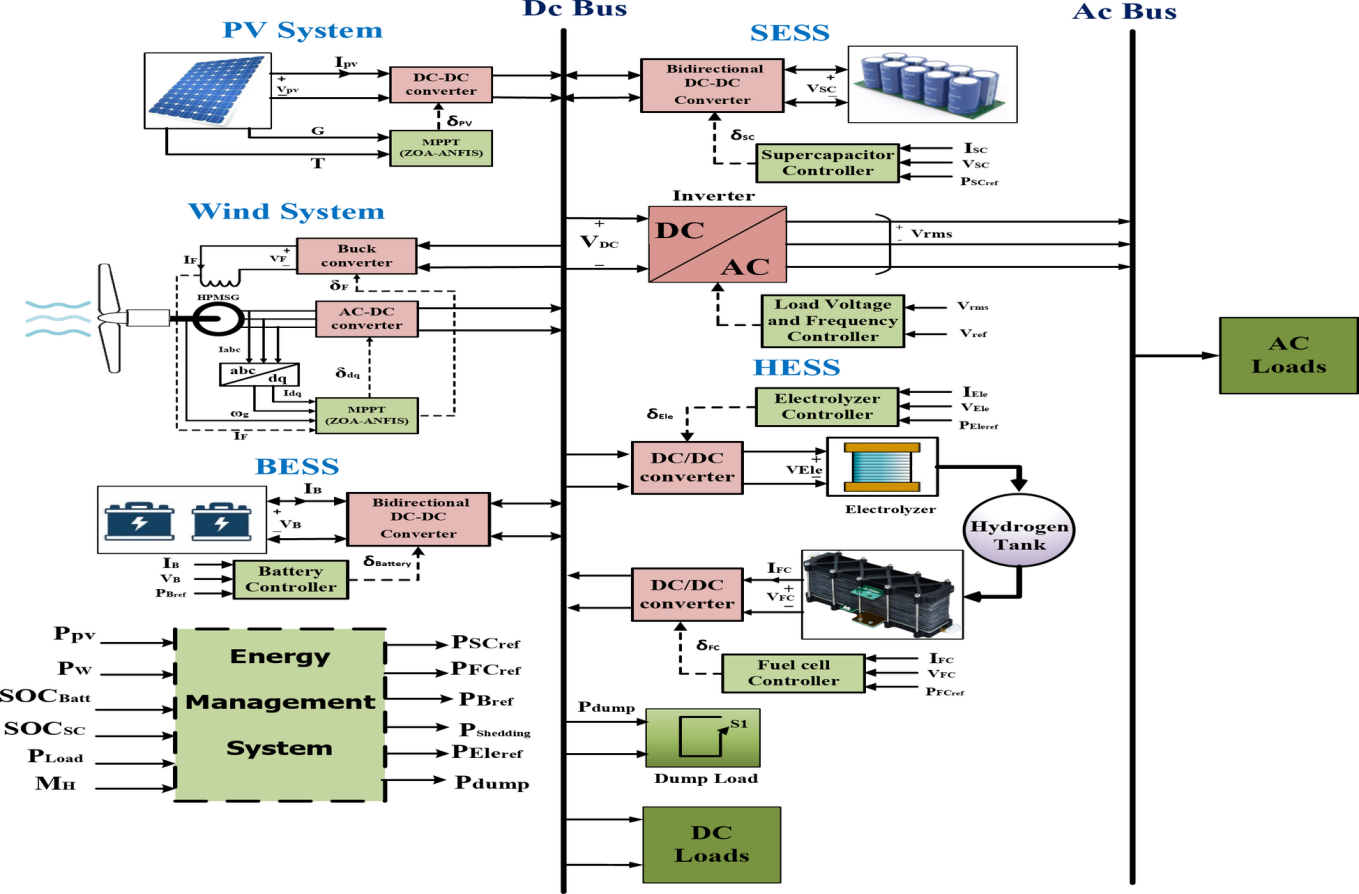
HRES architecture with a backup system.

The proposed HRES efficiently manages energy flow from PV and WTs sources, incorporating backup systems like FCs, SCs, and battery storage to ensure stable power supply to an isolated microgrid. The backup systems store surplus energy from renewables for times of low generation, with FCs providing a consistent energy source and SCs offering rapid energy bursts during demand spikes. These components are connected through a common DC bus, which also integrates DC-DC converters controlled by MPPT algorithms to optimize energy harvest from PV and WT sources as depicted in Fig. 1. A bidirectional converter connects the storage system to the DC bus, enabling efficient energy storage and transfer. An advanced energy management system, using optimization techniques, minimizes operational costs and reduces the LPSP. The system dynamically manages power flows based on real-time data from battery state-of-charge (SOC), renewable energy outputs, and hydrogen levels. This design ensures reliable, sustainable power for the isolated microgrid by efficiently handling energy production and demand fluctuations, enhancing resilience through integrated renewable and backup resources. The system’s configuration greatly improves overall reliability, ensuring it can seamlessly meet fluctuating electricity demand. Power electronic devices play a vital role in stabilizing and coordinating the operation of the system’s various components, enhancing both stability and efficiency. By effectively integrating renewable energy sources, energy storage, and power electronics, this hybrid system offers a promising solution to the challenges limiting the broader adoption of clean, distributed generation technologies. In addition to technical aspects, the system employs a sophisticated multi-objective optimization approach that addresses not only reliability but also economic and efficiency challenges. Through systematic optimization, the system is fine-tuned to achieve an optimal balance between reliability, economic feasibility, and overall efficiency.

### Wind generation system

The main component of a wind energy conversion system is WT, which harnesses kinetic energy from the wind. The wind energy conversion system power can be expressed as a function incorporating cut-in, cut-off and changing output with respect to wind velocity, as depicted in Eq. (1)^34–36^. This equation encapsulates the intricate process through which the system transforms kinetic energy from varying wind resources into a practical and usable electricity output.

1 $$\:{P}_{w}\:\left(v\right)=\:\left\{\begin{array}{c}\:\:\:\:\:\:\:\:0\:\:\:\:\:\:\:\:\:\:\:\:\:\:\:\:\:V\left(t\right)\le\:{V}_{in}\:or\:V\left(t\right)\ge\:{V}_{out}\\\:{P}_{r}\frac{V\left(t\right)-{V}_{in}}{{V}_{r}-{V}_{in}}\:\:\:\:\:\:\:\:\:\:\:\:\:\:{V}_{in}\le\:V\left(t\right)\le\:{V}_{r}\\\:{\:\:\:\:\:\:\:P}_{r}\:\:\:\:\:\:\:\:\:\:\:\:\:\:\:\:\:\:\:{\:\:\:\:\:\:\:\:\:\:\:\:V}_{r}\le\:V\left(t\right)\le\:{V}_{out}\end{array}\right.$$

Where, $\:\:{v}_{in}$represents Cut in speed of WT, $\:\:{v}_{out}$represents Cut off speed of WT, $\:\:{v}_{r}$ represents Rated speed of WT, $\:\:{v}_{t}$ represents actual speed of WT, $\:\:{P}_{r}$ represents rated power.

#### HEPMSM-based wind turbine

Hybrid Excited Permanent Magnet Synchronous Machines (HEPMSMs) are a promising technology that blends the strengths of Permanent Magnet Synchronous Machines (PMSMs) and Wound Field Synchronous Machines (WFSMs) to overcome their individual drawbacks. While PMSMs boast high efficiency and power density due to their permanent magnets, their fixed magnetic field hinders performance control in ever-changing conditions. Conversely, WFSMs provide flexibility through controlled magnetic fields, but their efficiency and power density suffer compared to PMSMs. HEPMSMs attempt to address these limitations by offering a hybrid solution that combines the advantages of both technologies^36–39^.

**Fig. 2:**
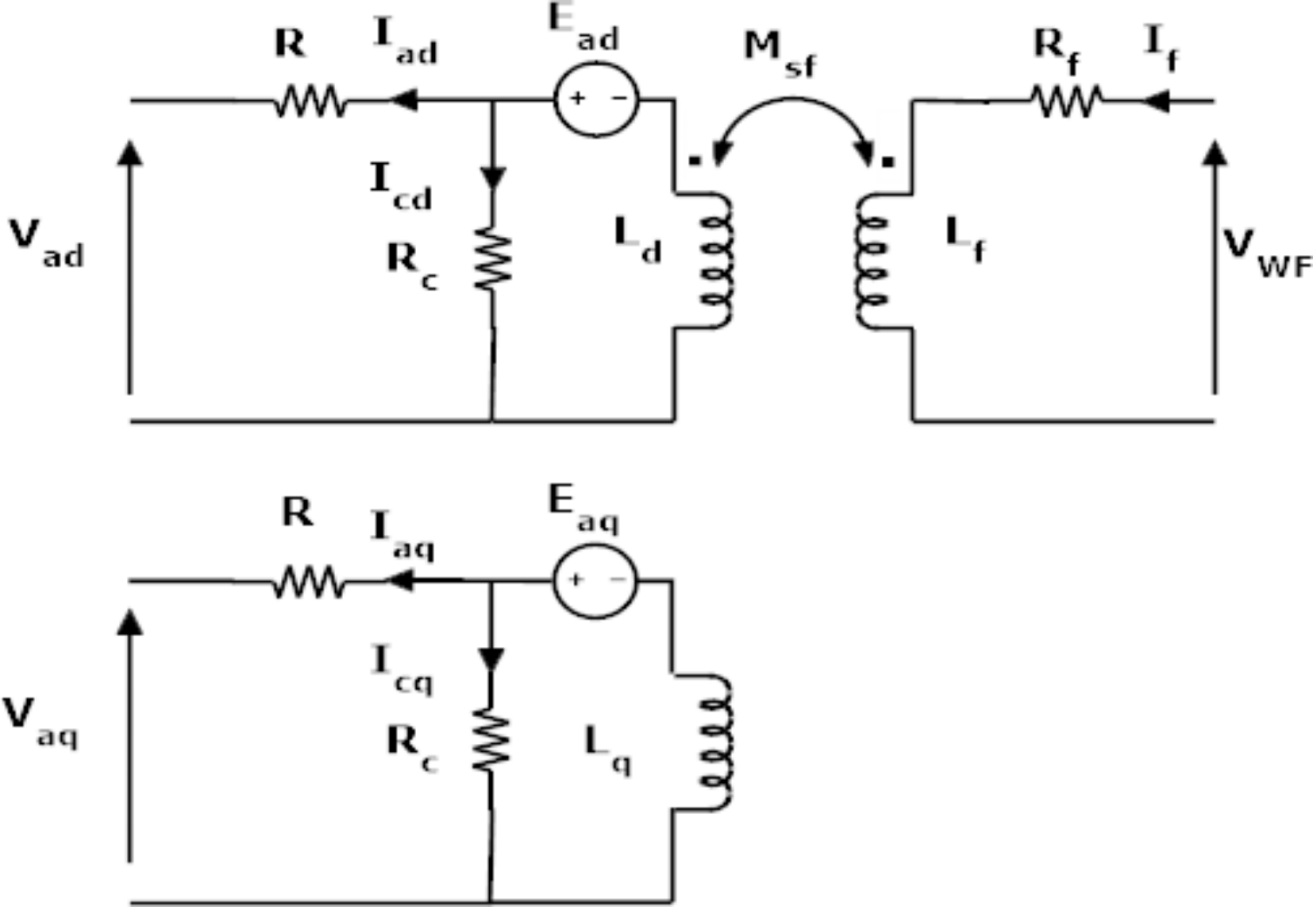
The d-q equivalent circuit of the proposed HEPMSG model.

WFSMs, on the other hand, offer the advantage of independent magnetic field control thanks to their stator windings. This flexibility makes them adaptable to diverse operating conditions. However, this adaptability comes at a cost. Compared to PMSMs, WFSMs struggle to achieve the same level of efficiency and power density, making them less ideal for applications where these factors are paramount, such as demanding high-performance compact designs^40,41^. Hybrid excitation permanent magnet machines (HEPMSMs) bridge the gap between PMSMs and WFSMs, offering the best of both machines. They retain the high efficiency and power density of PMSMs thanks to their permanent magnets, while incorporating the flexibility of WFSMs through an additional excitation system. This allows for precise control over the magnetic field, making HEPMSMs ideal for applications that demand both high performance and adaptable operation^42–44^. Figure 2 illustrates the d-q equivalent circuit of the proposed HEPMSM model. The equations below represent the relationship between the developed power, losses, and the currents flowing in the d-q axis and the field winding of HEPMSMs. It’s worth noting that since iron losses are negligible compared to copper losses, they are excluded from Eq. (3).

2 $$\:{P}_{d}({I}_{f},{I}_{ad},{I}_{aq})=\frac{3}{2}{\omega\:}_{s}({I}_{aq}\left({\lambda\:}_{PM}+\:{M}_{sf}{I}_{f}+{L}_{d}{I}_{ad}\right)\:+\left({L}_{q}{I}_{aq}\:\right){I}_{ad})$$

3 $$\:{P}_{loss}\left({I}_{f},{I}_{ad},{I}_{aq}\right)=\frac{3}{2}R\left({I}_{ad}^{2}+{I}_{aq}^{2}\right)+{I}_{f}^{2}{R}_{f})$$

Where, λ_PM_ and M_sf_ are the PM and WF excitation flux linkage, respectively, I_f_ is the excitation winding current, L_d_ and L_q_ are d- and q-axis inductance, respectively, I_ad_ and I_aq_ are the d- and q-axis armature current components. L_f_ is the excitation winding inductance.

### PV system

**Fig. 3:**
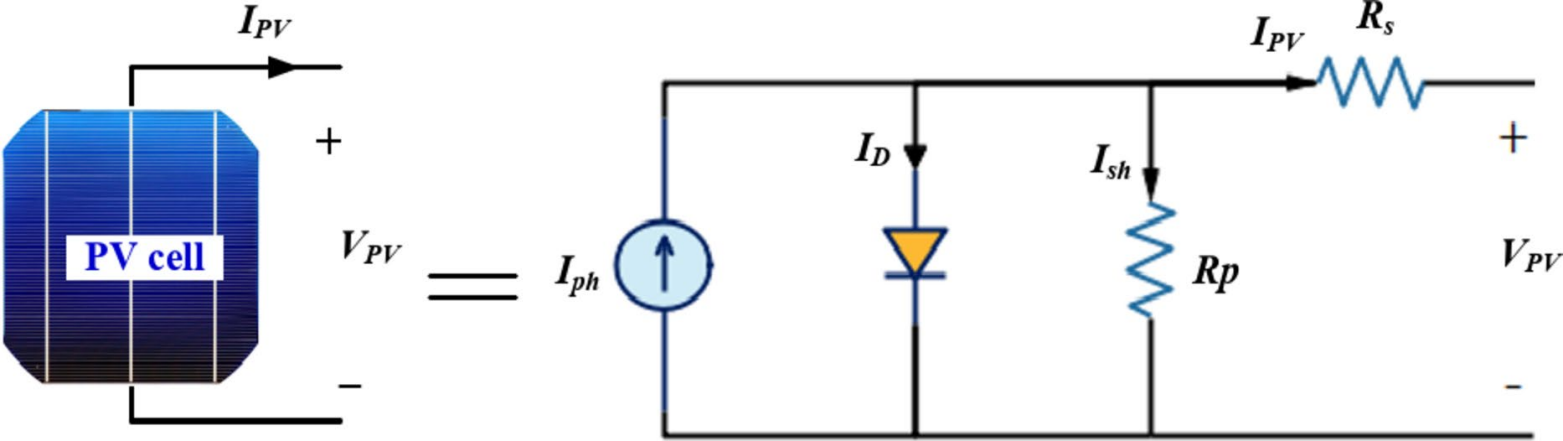
Single diode equivalent circuit of PV cell.

Once installed, Despite of the well-known PV system cost-effective over their operational lifespan, it is crucial to consider factors influencing the reliability these systems, such as weather conditions, geographical location, and sunlight availability and MPPT techniques The equivalent circuit of single-diode model for a photovoltaic cell is depicted in Fig. 3. The power produced by solar panels is primarily influenced by solar irradiation and is typically calculated using the following formulas^1^:

4 $$\:{I}_{PV}=G{N}_{p}{I}_{ph}-{I}_{o}\left({e}^{\left(\frac{q\left({N}_{p}{V}_{PV}\:+\:{N}_{s}\:{I}_{PV}{R}_{s}\right)}{akTNs{N}_{p}}\right)}-1\right)-\frac{{{N}_{p}V}_{PV}+{N}_{s}{I}_{PV}{R}_{s}}{{{N}_{s}R}_{sh}}\:\:\:\:$$

5 $$\:{P}_{pv}(V,{I}_{PV})=-V\hspace{0.17em}\left(\text{I}\text{o}\hspace{0.17em}\left({\text{e}}^{\frac{q\hspace{0.17em}\left({N}_{p}V+{\text{I}}_{pv}\hspace{0.17em}\text{N}\text{s}\hspace{0.17em}\text{R}\text{s}\right)}{A\hspace{0.17em}K{N}_{p}\hspace{0.17em}\text{N}\text{s}\hspace{0.17em}T}}-1\right)-G\hspace{0.17em}{I}_{ph}\hspace{0.17em}\text{N}\text{p}\hspace{0.17em}+\frac{\left(\text{N}\text{p}V+{\text{I}}_{pv}\hspace{0.17em}\text{N}\text{s}\hspace{0.17em}\text{R}\text{s}\right)}{\text{N}\text{s}\hspace{0.17em}\text{R}\text{p}}\right)$$

### Storage systems

The basic components of a hydrogen storage system are given as:

#### Electrolyzer

Applying direct current to two electrodes immersed in water (H_2_O), results in separation of water molecules into their constituent elements hydrogen (H_2_) and oxygen (O_2_) as in the following Eq. (1):

6 $$H_{2} O\mathop \to \limits^{{heat}} ~2H^{ + } + \frac{1}{2}O_{2}^{{2 - }}$$

The produced hydrogen gas is typically collected and stored in a dedicated tank. To optimize the storage capacity and efficiency of the hydrogen storage system, the hydrogen is typically stored at a higher pressure within the main tank.

For the system proposed here, hydrogen generated through electrolysis is fed directly into the main storage tank. The power output transferred from the electrolyzer to the hydrogen tank can be expressed by the following equation^45^:

7 $$\:{P}_{ELE/HT}=\:{\eta\:}_{ELE}\:.\:{P}_{Sur/ELE}\:$$

Where, $\:{\eta\:}_{ELE}$ denotes the efficiency of an electrolyzer and $\:{P}_{Sur/ELE}$ represents the electrical energy feeding the electrolyzer from renewable sources.

#### Fuel cell

FCs operate in a reverse manner compared to the electrolyzer. It combines the stored hydrogen with oxygen to produce electricity as its primary output, accompanied by the generation of water and heat as byproducts. This electricity generation is achieved through the ionization of hydrogen, resulting in the release of electrons as in the following equations:

8 $$\:{H}_{2}\:\to\:2\:{H}^{+}+2\:{e}^{-}$$

9 $$\:{O}_{2}+\:2\:{e}^{-}\:\to\:\:{O}_{2}^{2-}$$

10 $$2H^{ + } + \frac{1}{2}O_{2}^{{2 - }} ~ \to ~H_{2} O + Heat$$

For this specific system, a proton-exchange membrane fuel cell (PEMFC) is employed due to its notable advantages, including rapid startup times. The characteristics of PEMFCs make them highly suitable for this particular application.

The mathematical modeling of the PEMFC involves Eq. (11) that establishes a relationship between its power output and the power transferred from the hydrogen storage system^45^. taking into consideration the efficiency of the fuel cell. This efficiency value reflects the typical performance of PEMFCs and their ability to convert a significant portion of the chemical energy contained in the hydrogen fuel into usable electrical energy.

11 $$\:{P}_{FC}\:\left(t\right)={\:\eta\:}_{FC}*{P}_{strg-FC}\:\left(t\right)$$

Where, P_FC_(t) is the generated electrical power from PEMFC. $\:{\:\eta\:}_{FC}\:$is the overall efficiency of fuel cell and $\:{P}_{strg-FC}\:\left(t\right)$ is the power transferred from hydrogen storage tank to Fuel cell.

The model characterizes the PEMFC’s role in converting the chemical energy stored in hydrogen back into electrical energy on demand through electrochemical reactions with oxygen, while also describing its expected power generation performance based on efficiency and fuel supply parameters.

#### Hydrogen tank

The hydrogen tank serves as the primary storage medium for hydrogen gas. It is designed to safely store and contain hydrogen under specific conditions, including pressure and temperature. The tank is constructed using materials that ensure the integrity and durability of the stored hydrogen, preventing leakage and maintaining its purity. Different types of hydrogen tanks are available, such as high-pressure (compressed gas) tanks or cryogenic tanks for storing liquid hydrogen. The choice of tank depends on factors such as storage capacity, weight, safety considerations, and the specific requirements of the application. The tank capacity is determined by following equation considering factors like the mass of electrolyzed hydrogen and its pressure/density characteristics:

12 $$\:{V}_{{H}_{2}}=\:\frac{{m}_{{H}_{2}.EL}\:x\:{M}_{HT}}{{P}_{{H}_{2}}\:x\:{\rho\:}_{{H}_{2}}}$$

The hydrogen storage used in the proposed system is mathematically modeled using the energy it is storing inside at time t which is shown by E_H2strg_ (t) and computed as follows^45^:

13 $$\:{E}_{H2strg}\:\left(t\:\right)=\:{E}_{H2strg}\:\left(t-1\right)+\:\:dt\:({P}_{eLe/strg}\:\left(t\right)\:-{P}_{\:str/FC}\:\:(t)/{\eta\:}_{strg})$$

On the contrary, when there is a shortage of power generated from solar and wind energy sources, the stored hydrogen is used to generate electricity through FCs. In this case, the amount of hydrogen stored in the tank can be determined as follows:

14 $$\:{M}_{H2}\left(t\right)=\:\frac{{E}_{H2strg}\:\left(t\:\right)}{{H}_{Hv}}\:\:\:\:\:\:\:\:\:\:\:\:\:\:\:\:\:\:\:\:\:\:\:\:\:\:\:\:\:\:\:\:\:\:\:\:\:\:\:\:\:\:\:\:\:\:\:\:\:\:\:\:\:\:\:\:\:\:\:\:\:\:\:\:\:\:\:\:\:\:\:\:\:\:\:$$

Where, H_Hv_ denotes the higher heat value of the stored hydrogen gas (kWh/kg).

#### Battery storage system

As a backup power source, the Battery Energy Storage System (BESS) is essential to maintaining stability and enhancing the dependability of a self-contained microgrid. This becomes particularly vital due to the variable nature of renewable energy sources (RESs) whose production is subject to fluctuations based on weather conditions. By relying on the BESS, the microgrid effectively adapts to these dynamic changes, providing a reliable and continuous power supply. One significant advantage of incorporating the BESS is its ability to minimize energy losses. This is achieved by efficiently managing surplus energy generated by Photovoltaic (PV) and Wind Energy Conversion Systems (WECS). Instead of allowing the excess energy to be directed solely towards the Distributed Load (DL), the BESS actively absorbs and stores the surplus power. This process ensures that the energy generated by the RESs is optimally utilized, reducing wastage, and enhancing overall system efficiency.

The charging and discharging operations of the BESS are intricately linked to the amount of energy generated. When there is an excess output of power from the RESs, the surplus energy is intelligently directed towards charging the BESS, effectively storing it for later use. This allows for efficient energy management, ensuring that no renewable energy goes to waste. By utilizing the BESS as an intermediary energy buffer, the microgrid can effectively balance supply and demand, optimizing the utilization of available resources. The stored energy, accumulated during the charging process, is expressed as follows^46,47^:

15 $$\:{E}_{batt}\left(t\right)=\:{E}_{batt}\left(t-1\right).\left(1-\sigma\:\right)+{{\upeta\:}}_{batt}\:\:{P}_{batt}\left(t\right).dt$$

During discharge, the stored energy in the BESS is estimated as follows:

16 $$\:{E}_{batt}\left(t\right)=\:{E}_{batt}\left(t-1\right).\left(1-\sigma\:\right)-{{\upeta\:}}_{batt}\:\:{P}_{batt}\left(t\right).dt$$

The energy stored in BESS depends on these constraints:

17 $$\:{E}_{batt-min}\:\le\:{E}_{batt}\left(t\right)\le\:\:{E}_{batt-max}$$

18 $$\:{E}_{batt-min}\:=\:\text{D}\text{O}\text{D}.\:{E}_{batt-nom}$$

19 $$\:{P}_{batt}^{min}\:<\:{P}_{batt}\left(t\right)<\:{P}_{batt}^{max}$$

Where E_batt(t)_ represents the energy stored in the battery at any given time *t*, η_batt_ is the battery efficiency, DOD is the depth of discharge which is the percentage of battery capacity extracted during discharge, E_batt−min_ and E_batt−max_ define the minimum and maximum allowable energy that can be stored in the battery, and σ is the self-discharge rate which refers to the percentage of battery capacity that will dissipate over time even when the battery is not being charged or discharged.

#### Supercapacitor storage system

In recent times, significant advancements have been witnessed in BESS, coinciding with a surge in interest surrounding SCs. Laboratories and researchers are actively involved in driving the progress of these technologies, focusing on areas such as size reduction, efficiency enhancement, energy quality improvement, and environmental sustainability. Overcoming these challenges is pivotal for the continued evolution of energy storage systems. The notable strides observed in BESS and the increased attention towards SCs stem from the quest for technologies that not only deliver exceptional performance but also address critical concerns like size, efficiency, and environmental impact. Researchers are tirelessly working to push the boundaries, aiming to make these storage technologies more efficient, compact, and environmentally friendly. Supercapacitor Energy Storage Systems (SESS) have emerged as a promising technology that effectively bridges the gap between traditional batteries and capacitors. SESS sets itself apart from conventional capacitors by not only storing larger quantities of energy but also providing higher energy outputs compared to typical batteries. These distinctive advantages, combined with long-term stability, contribute to the growing appeal of SESS for various energy storage applications. One crucial aspect of SCs is the estimation of their output voltage, which can be calculated using the following equation^48^:

20 $$\:{V}_{SC}=\:\frac{{N}_{SC}\:{Q}_{ec}\:d}{{N}_{pc\:}{N}_{e}\:{\epsilon\:\epsilon\:}_{0}\:{A}_{i}}+\:\frac{2\:{N}_{e}{N}_{SC}{R}_{d}T}{F}{sinh}^{-1}\left(\frac{{Q}_{ec}}{{N}_{pc}\:{N}_{e}^{2}\:{A}_{i}\:\sqrt{8\:{R}_{d}\:T\:{\epsilon\:\epsilon\:}_{0}\:{C}_{m}\:}}\right)-\:{R}_{SC}\:.\:{i}_{SC}\:$$

21 $$\:{Q}_{ec}\:=\:\int\:{i}_{SC}\:dt$$

22 $$\:{Q}_{ec}\:=\:\int\:{i}_{self\_dis}\:dt$$

Where,

23 $$\:{i}_{self\_dis}\:=\:\left\{\begin{array}{c}\frac{{C}_{T}{\alpha\:}_{1}}{1+s{R}_{SC}{C}_{T}}\:if\:t-\:{t}_{oc}\le\:\:{t}_{3}\\\:\frac{{C}_{T}{\alpha\:}_{2}}{1+s{R}_{SC}{C}_{T}}\:if\:{t}_{3}\:<t-\:{t}_{oc}\le\:\:{t}_{4}\\\:\frac{{C}_{T}{\alpha\:}_{3}}{1+s{R}_{SC}{C}_{T}}\:if\:t-\:{t}_{oc}\ge\:\:{t}_{4}\end{array}\:\right.$$

The energy stored in the SCs can be expressed by the following equation^49^:

24 $$\:{E}_{SC}\left(t+\varDelta\:t\right)=\:{E}_{SC}\left(t\right)+\:{\eta\:}_{SC}\:\varDelta\:t\:{P}_{SC}\left(t\right)$$

Where,

25 $$\:{E}_{SC}^{min}\le\:{E}_{SC}\left(t\right)\le\:{E}_{SC}^{max}\:$$

26 $$\:{P}_{sc}^{min}\le\:{P}_{SC}\left(t\right)\le\:{P}_{SC}^{max}$$

Where, N_SC_ is the number of series supercapacitors, N_pc_ is the number of parallel supercapacitors, Q_ec_ is the electric charge, N_e_ is the number of layers of electrodes, ε_0_ refers to permittivity of free space, while A_i_ is the electrodes and electrolyte interfacial area, R_d_ is the molecular radius, T is the operating temperature, F refers to the Faraday constant, C_m_ is the molar concentration, R_SC_ is the total resistance of the used supercapacitors, i_SC_ is the output current of supercapacitor, α_i_ is the voltage rates of change, C_T_ is the total capacitance, s is the complex frequency, η_SC_ - charging/discharging efficiency, ξ - self-discharge rate, P_SC_ - power supplied or drawn to or from supercapacitors, and Np - number of cells connected in parallel. $\:{P}_{sc}^{min}$ and $\:{P}_{SC}^{max}$ refer to the maximum and minimum allowable power can be drawn to or from supercapacitors. $\:{E}_{SC}^{min}$ and $\:{E}_{SC}^{max}\:$ define the minimum and maximum allowable energy that can be stored in the supercapacitors. Together, these parameters define the operational, electrical, and efficiency characteristics, as well as power capabilities of supercapacitors in diverse applications.

Were,

#### Conversion stage

The hybrid system under consideration comprises an inverter and a rectifier. The role of the rectifier is to convert the AC power generated by the WT into DC power, facilitating its utilization in the hydrogen, battery, and supercapacitor energy storage systems. The modeling of the rectifier involves the use of the following equations:

27 $$\:{P}_{rec\_out}\left(t\right)=\:{\eta\:}_{rec}*{P}_{re{c}_{in}}\left(t\right)$$

While load is fed from inverter the following equation represents the modelling of inverter:

28 $$\:{P}_{in{v}_{out}}\left(t\right)=\:{\eta\:}_{inv}*{P}_{in{v}_{in}}\left(t\right)$$

As $\:{P}_{rec\_out}\left(t\right)$ and $\:{P}_{in{v}_{out}}\left(t\right)$ are the output power extracted from rectifier and inverter respectively. $\:{\eta\:}_{rec}$ and $\:{\eta\:}_{inv}$

Are the efficiency of rectifier and inverter respectively. While the input power for rectifier and inverter is denoted by$\:{\:P}_{re{c}_{in}}\left(t\right)$ and $\:{P}_{in{v}_{in}}\left(t\right)$.

## EM control architecture

**Fig. 4:**
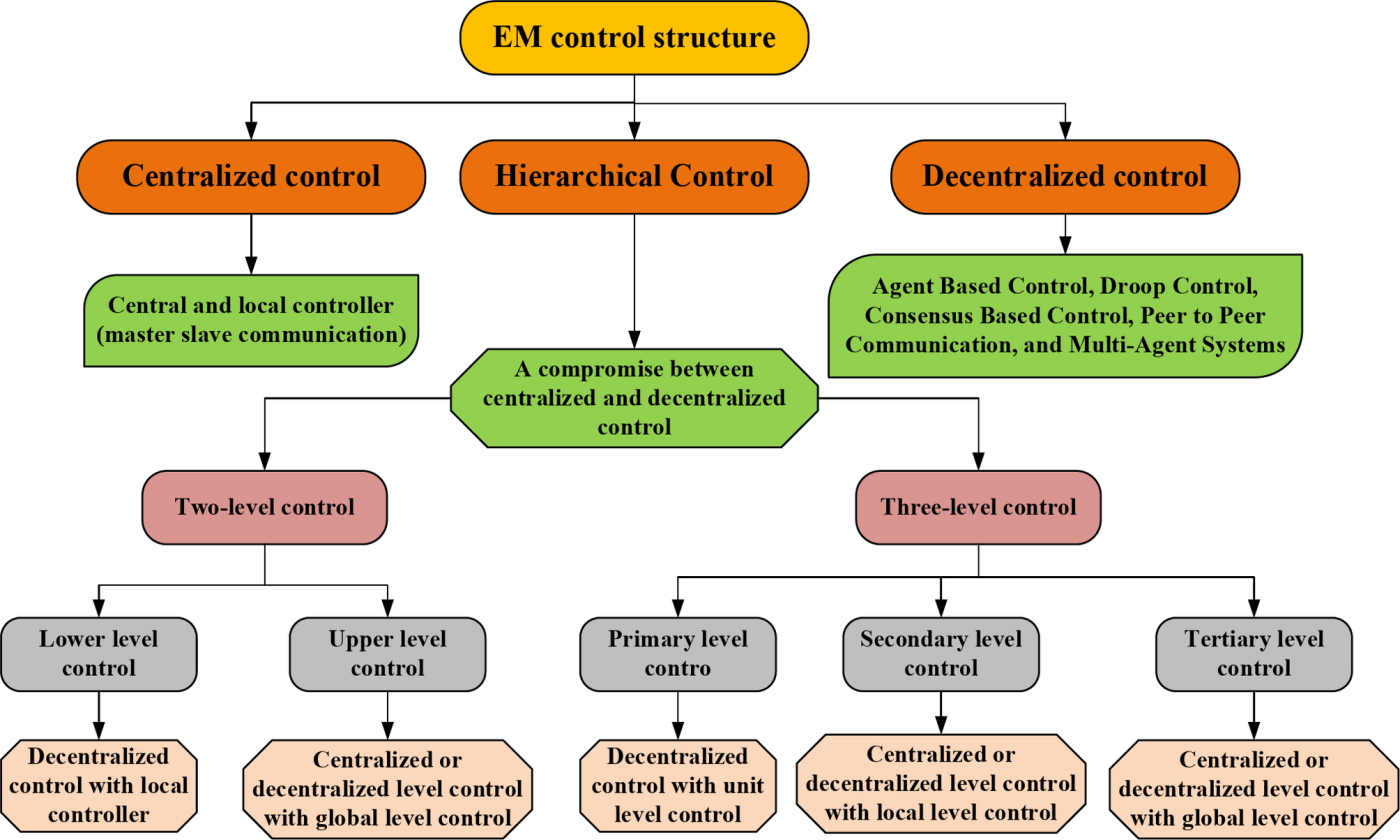
Categories of energy management (EM) control strategies.

**Fig. 5:**
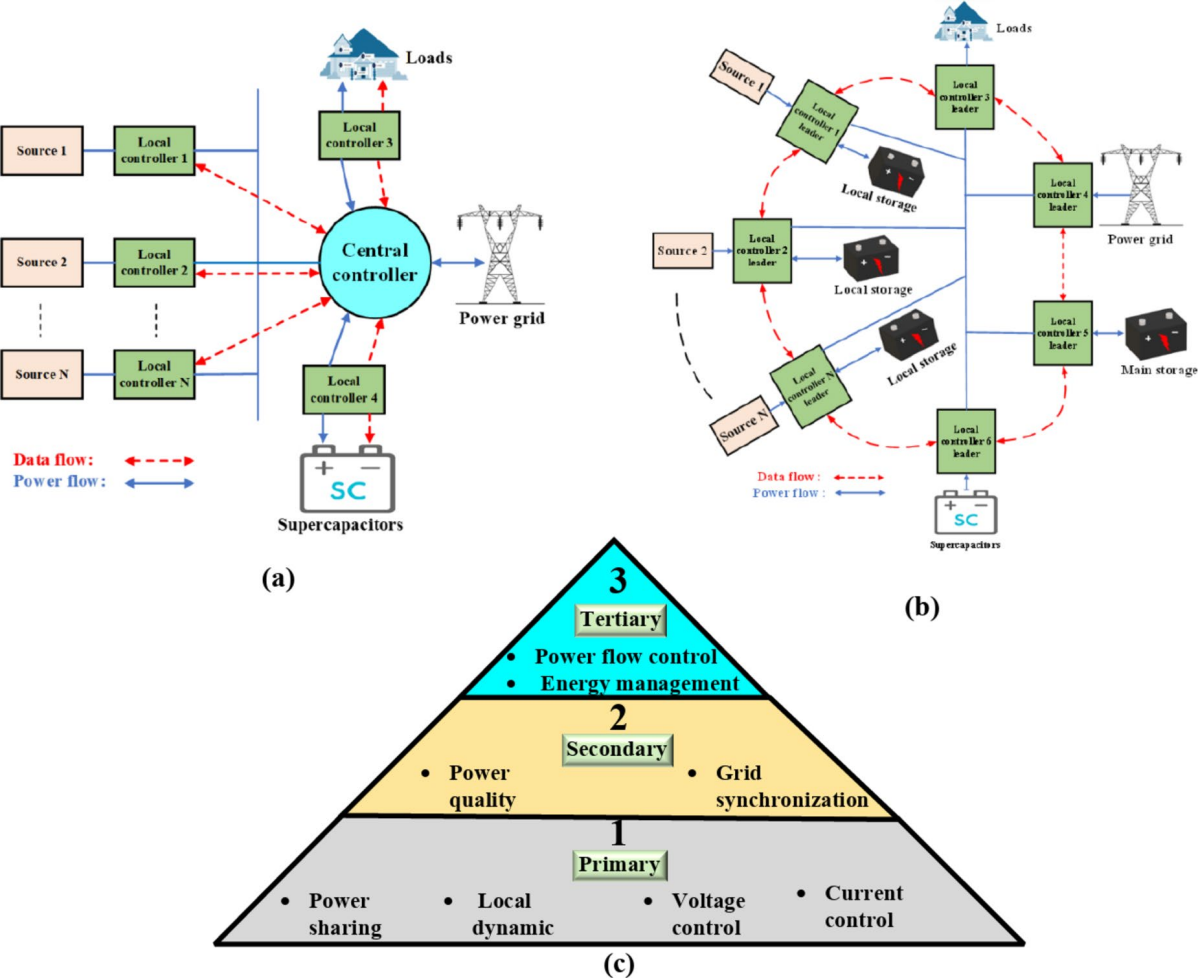
(**a**) Centralized control strategy, (**b**) decentralized control strategy, (**c**) hierarchical control strategy.

Efficient energy management is crucial across various systems, including industrial processes, smart grids, and renewable energy systems, due to the essential relationship between resource use and operational performance. In these settings, control architectures are key to optimizing energy consumption, improving overall efficiency, and ensuring reliable system operation. These control structures function as intelligent frameworks, managing communication and decision-making to adapt to changing conditions. By regulating the interactions between components, such as sensors and controllers, they are vital for achieving sustainability goals, reducing waste, and strengthening the resilience of critical infrastructure in a dynamic energy environment. To optimize processes and boost efficiency, different control architectures are utilized in energy management, with three common types being hierarchical, decentralized, and centralized control, as illustrated in Fig. 4^50^. Centralized control architectures consolidate decision-making authority in a central entity, often a controller or computer system^50^. The centralized control strategy is shown in Fig. 5a. This central authority processes information from sensors, making decisions communicated throughout the system. Communication is typically radial, with information flowing to and from the central controller. Advantages include global optimization, efficient resource allocation, and simplified system integration. However, vulnerabilities exist in terms of potential single points of failure and communication bottlenecks. While centralized control brings benefits like global optimization and unified decision-making, it also poses limitations, including scalability challenges and increased vulnerability to cybersecurity threats. Careful consideration is necessary to balance these aspects and assess the suitability of centralized control architectures for specific systems.

In decentralized control architectures, decision-making is dispersed among different entities or subsystems, each possessing a degree of autonomy to make local decisions based on its observations and objectives. The decentralized control strategy is shown in Fig. 5b. Communication in decentralized control usually takes a peer-to-peer approach, enabling direct information exchange among subsystems. This design fosters flexibility and fault tolerance, allowing subsystems to operate independently, making it well-suited for large-scale and distributed systems. Despite its advantages, challenges may emerge in maintaining coherent decision-making across the entire system. Decentralized control offers benefits like enhanced flexibility, fault tolerance, and localized decision-making, empowering subsystems to autonomously adapt and bolstering overall system resilience. However, challenges encompass effective coordination and communication between entities, ensuring consistency and decision integration, managing design complexities, and addressing security concerns. While decentralized control supports scalability and swift responses to local changes, meticulous consideration and management are crucial for navigating subsystem interactions and optimizing system performance.

Hierarchical control structures employ layered decision-making processes, featuring multiple levels of control, each assigned specific functions within the system as shown in Fig. 5c. Decision-making authority cascades from higher to lower levels, addressing distinct aspects of energy management. The communication structures are vertically organized, facilitating efficient information flow between different levels. This architecture proves advantageous in complex systems, fostering a clear division of tasks and responsibilities that contributes to enhanced system integration and adaptability. In energy management, hierarchical control structures follow a tiered approach, where higher levels concentrate on overarching goals, including goal setting, long-term planning, and resource allocation. Meanwhile, lower levels handle finer details, encompassing real-time control and feedback. Tasks are adeptly divided among these layers to prevent overload, ensuring a well-coordinated workflow. Responsibilities are allocated based on expertise, with high-level layers managing policies, intermediate layers overseeing coordination, and control layers ensuring immediate responsiveness. This streamlined approach enhances overall efficiency, facilitating adaptability to system dynamics and scalability across varied complexities, making hierarchical control structures indispensable for optimizing energy management in diverse scenarios.

## Proposed EMS and optimizer

Traditionally, an Energy Management System (EMS) optimizes power flow to meet the energy demands of a microgrid, balancing the energy produced by photovoltaic (PV) and wind sources with the energy consumed by the system. The main objective of the proposed EMS is to optimize power flow, reducing both operational and maintenance costs, as well as the Loss of Power Supply Probability (LPSP) for the microgrid. The EMS operates within a hybrid system that integrates PV and wind energy sources, supported by three energy storage systems: battery, supercapacitor, and hydrogen storage. It actively manages the State of Charge (SOC) of each storage system to ensure their optimal use and efficiency. Table 1 outlines the operating modes and availability of each energy source in the microgrid. During unexpected fluctuations, grid failures, or other emergencies, the EMS switches to backup power sources, using the stored energy in the battery, supercapacitor, and hydrogen systems to maintain power continuity and stability. The EMS ensures a smooth transition to backup power, minimizing downtime and disruptions to critical loads. The performance of this EMS is evaluated across various SOC levels of the energy storage systems, which can be categorized into three operational modes:

- 1st mode, the power demand is fulfilled by both primary and backup energy sources. The battery’s charge level is maintained between 20% and 80%, the supercapacitor’s charge level is between 50% and 90%, and the hydrogen tank contains between 1 kg and 5 kg to supply the fuel cell with the required hydrogen for power generation. During this mode, there is no need for load shedding or the use of a dump load, as any shortfall in power can be compensated for by the energy storage system when primary sources alone are insufficient to meet the load demand. Moreover, in instances of excess power from primary sources, the Energy Management System (EMS) redirects this surplus power to the energy storage elements, considering constraints related to charging and discharging. This process takes into account the economic, reliability, and flexibility aspects of the HRES.
- 2nd mode, where the demand for power exceeds the generated capacity as all energy storage systems are under the charge limitations so it cannot provide power for load, the proposed EMS demonstrates its adaptive capabilities by swiftly initiating load shedding decisions with pinpoint accuracy, effectively optimizing power distribution, and ensuring continued operational stability. This dynamic response mechanism plays a pivotal role in mitigating potential disruptions and maintaining uninterrupted power supply, even during periods of heightened demand.
- 3rd mode, when the generated power surpasses the immediate load demand and the storage system components reach full capacity, the proposed EMS seamlessly redirects excess power to auxiliary loads (dummy loads), thereby preventing overcharging of storage elements and bolstering grid stability. This innovative feature not only optimizes energy utilization but also contributes to the overall resilience and sustainability of microgrid operations.

**Table 1:** Operation modes of the proposed EMS.

Mode	Energy storage configuration	Energy storage system statement		
		Hydrogen tank level	SOC-Batt	SOC-Sc
1	Fuel cell, batteries, and SCs	✓	✓	✓
2	No energy storage options utilized	✘	✘	✘
3	All energy storage elements with fully charged states	Full	Full	Full

The honey badger, a mammal renowned for its bravery, inhabits regions such as semi-deserts and African rainforests. Through observing their behavior while foraging for food, a simple optimization mechanism called the HBA was developed. Honey badgers employ two primary strategies to find food: using their strong sense of smell to locate prey or digging to uncover it. Additionally, they can track the locations of beehives by following honey guide birds. These two foraging methods are termed the “digging stage” and the “honey stage,” respectively. The honey badgers adjust their speed based on the strength of the scent they detect, moving quickly when the scent is strong and more slowly when it is faint. The core control procedures and equations of the HBA are derived from these behaviors and can be summarized as follows:^51^:

- Initialization stage of the HBA: The number (*N*) and locations of honey badgers are initialized according to Eq. (16).

29 $$\:{x}_{i}\:=\:{lb}_{i}\:+\:{r}_{1}\:\times\:\:\left({ub}_{i}\:-\:{lb}_{i}\right)\:\:\:\:\:\:\:\:\:\:\:\:\:\:\:\:\:\:\:\:\:\:\:\:\:\:\:\:\:\:\:\:\:\:\:\:\:\:\:\:\:\:\:\:\:\:\:\:\:\:\:\:\:\:\:\:\:\:\:\:\:\:\:\:\:\:\:\:\:\:\:\:\:\:\:\:\:\:\:\:\:\:\:\:\:\:$$

Where; *r*_*1*_ is a random value ranging from 0 to 1, the lower and upper bounds are expressed in *lb*_*i*_ and *ub*_*i*_, respectively, and *x*_*i*_ is i^th^ location of the honey badger that provides a candidate solution in its population group *N*. honey badger position referring to a candidate solution in a population of N, while and are respectively lower and upper bounds of the search domain.

(b) Determine the intensity (I_i_): The intensity of the prey scent (*I*_*i*_) depends on the location of the i^th^ honey badger, the farther the honey badger is from its prey, the less intense it is and vice versa. If the intensity of the smell is high, the honey badger will move very quickly, and the lower the intensity, the slower the speed. The relationship between distance and intensity can be represented by applying the inverse square law as in Eq. (30).

30 $$\:\:\left\{\begin{array}{c}{I}_{i}={r}_{2}\times\:\frac{S}{{4\pi\:d}_{i}^{2}}\\\:S={\left({x}_{i}-{x}_{i+1}\right)}^{2}\\\:{d}_{i}={x}_{prey}-{x}_{i}\end{array}\right.\:\:\:\:\:\:\:\:\:\:\:\:\:\:\:\:\:\:\:\:\:\:\:\:\:\:\:\:\:\:\:\:\:\:\:\:\:\:\:\:\:\:\:\:\:\:\:\:\:\:\:\:\:\:\:\:\:\:\:\:\:\:\:\:\:\:\:\:\:\:\:\:\:\:\:\:\:\:\:\:\:\:\:\:\:\:\:\:\:\:\:\:\:\:\:\:\:\:\:\:\:\:\:\:\:\:\:\:\:\:\:\:\:\:$$

Where, *r*_*2*_ is a random value ranging from 0 to 1, *S* is the concentration intensity which reflects the location of the prey (location of prey), *x*_*prey*_ is the location of the prey (best location), and *d*_*i*_ is the separation between the i^th^ badger and its prey.

(c) Updating of density factor (α): To achieve a smooth transition from the exploration stage to the exploitation stage, the random distribution that changes with time is controlled by the density factor (decreasing factor) (*α*). *α* is updated by using the mathematical Eq. (31) that ensures a gradual decrease with each iteration and therefore the extent of randomness can be reduced over time.

31 $$\:\alpha\:=C\times\:{e}^{\left(\frac{-t}{{t}_{max}}\right)}\:\:\:\:\:\:\:\:\:\:\:\:\:\:\:\:\:\:\:\:\:\:\:\:\:\:\:\:\:\:\:\:\:\:\:\:\:\:\:\:\:\:\:\:\:\:\:\:\:\:\:\:\:\:\:\:\:\:\:\:\:\:\:\:\:\:\:\:\:\:\:\:\:\:\:\:\:\:\:\:\:\:\:\:\:\:\:\:\:\:\:\:\:\:\:\:\:\:\:\:\:\:\:\:\:\:\:\:\:\:\:$$

Where, *t*_*max*_ is the maximum number of iterations, and *C* is a constant larger than 1.

(d) Escaping From Local Optimum: This procedure and the next two are used to prevent being stuck in local optima areas. This algorithm adds a flag F that modifies the search direction, providing agents more opportunities to properly explore the search field.
(e) Updating the positions of agents: The “digging stage” and the “honey stage” are two detached steps that form the position updating process (*x*_*new*_) for HBA. These stages have various functions, which are described in detail below:

- Digging stage: The action pattern of the honey badger and the cardioid form are very similar; in addition, the cardioid motion can be simulated by the relationship described in Eq. (32). During this stage, the honey badger specifically makes decisions based on this equation. 

32 $$\:{x}_{new}=\:{x}_{prey}\:+\:F\:\beta\:\:{I}_{i}\:{x}_{prey}\:+\:\left(F\:{r}_{3}\:\alpha\:\:{d}_{i}\times\:\left|{cos}\left(2\pi\:{r}_{4}\right)\times\:\left[1\:-{cos}\left(2\pi\:{r}_{5}\right)\right]\right|\right)\:\:\:\:\:\:\:\:\:\:\:\:\:\:\:\:\:\:\:\:\:\:\:\:\:\:\:\:\:\:\:\:\:\:\:\:\:\:\:\:\:\:\:$$

Where, *β* (≥ 1) is the capability of the honey badger to obtain food, and *r*_*3*_, *r*_*4*_, *r*_*5*_, *r*_*6*_, and *r*_*7*_ are random values ranging from 0 to1.

The flag F changes the direction of the search and can be calculated as in Eq. (33):

33 $$\:F=\left\{\:\begin{array}{c}1\:\:\:\:\:\:\:\:\:\:\:\:\:\:\:\:\:\:\:\:\:\:if\:{r}_{6}\le\:0.5\\\:-1\:\:\:\:\:\:\:\:\:\:\:\:\:\:\:\:\:\:\:\:\:else\:\:\:\:\:\:\:\:\:\:\:\:\:\:\:\:\:\end{array}\right.\:\:\:\:\:\:\:\:\:\:\:\:\:\:\:\:\:\:\:\:\:\:\:\:\:\:\:\:\:\:\:\:\:\:\:\:\:\:\:\:\:\:\:\:\:\:\:\:\:\:\:\:\:\:\:\:\:\:\:\:\:\:\:\:\:\:\:\:\:\:\:\:\:\:\:\:\:\:\:\:\:\:\:\:\:\:$$

- Honey stage: The case in which the honey guide bird guides the honey badger to the beehive can be simulated using Eq. (34).

34 $$\:{x}_{new}\:=\:{x}_{prey}+\:F\:{r}_{7}\:\alpha\:{\:d}_{i}\:\:\:\:\:\:\:\:\:\:\:\:\:\:\:\:\:\:\:\:\:\:\:\:\:\:\:\:\:\:\:\:\:\:\:\:\:\:\:\:\:\:\:\:\:\:\:\:\:\:\:\:\:\:\:\:\:\:\:\:\:\:\:\:\:\:\:\:\:\:\:\:\:\:\:\:\:\:\:\:\:\:\:\:\:\:\:\:\:\:\:\:\:\:\:\:\:\:\:\:\:\:\:$$

**Fig. 6:**
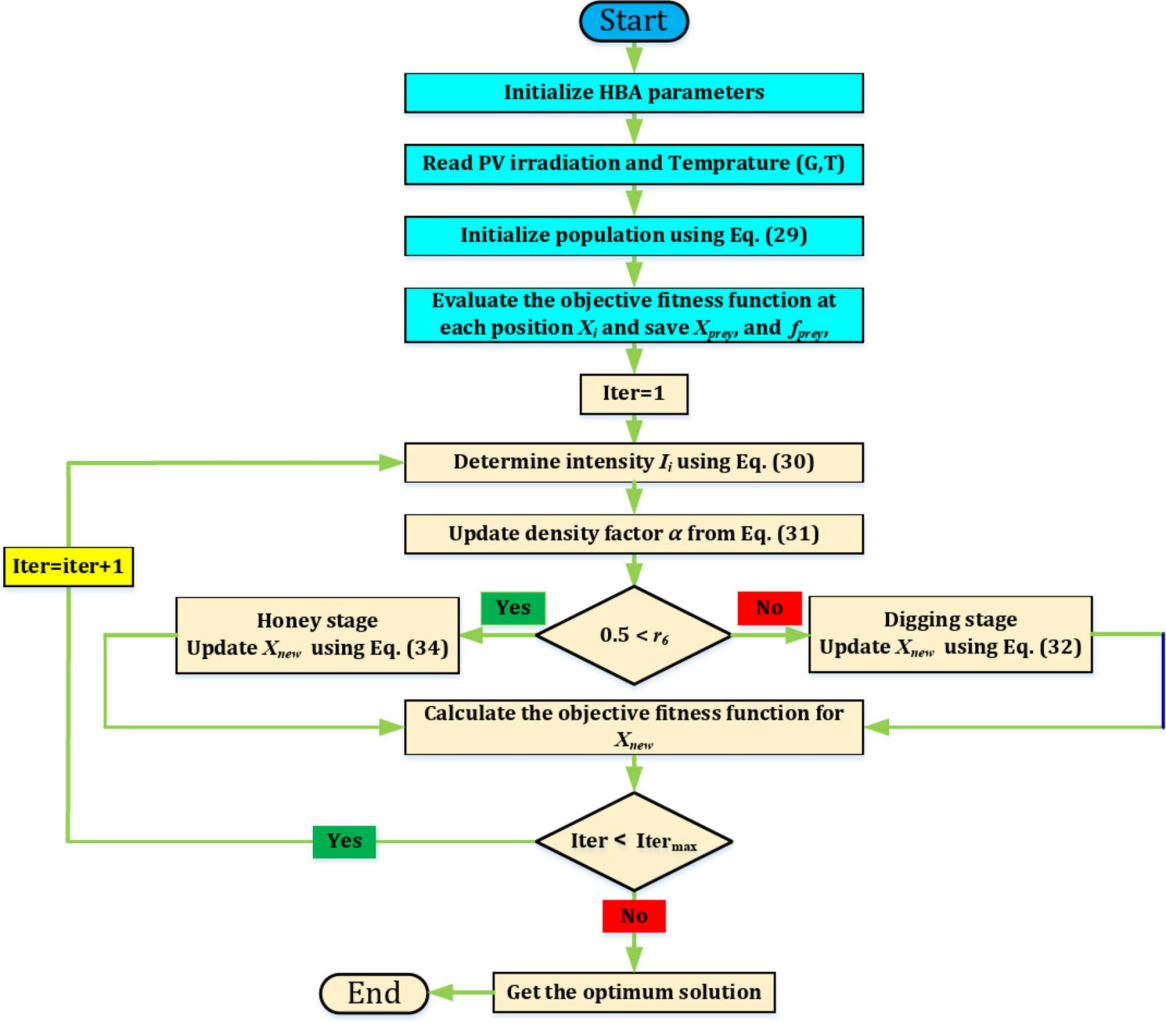
Flowchart for the HBA.

The honey badger exploits distance information (*d*_*i*_) to guide its search when it is near its prey location (*x*_*prey*_). Moreover, its behavior is affected by the time-varying searching pattern (*α*), however it can encounter disturbances F while searching. A summary of the steps describing the HBA is presented as depicted in Fig. 6.

## Formulation of the system problems and optimal operational approach for ESS

### Objective function

The main goal of this study is to minimize a composite objective function that combines two key metrics: operating and maintenance cost (O&M) and LPSP. The objective function is formulated as follows:

35 $$\:Objective\:Function\:\left(OF\right)={\theta\:}_{O\&M}+LPSP$$

Where, $\:{\theta\:}_{O\&M}$ represents the operating and maintenance cost per hour.

The weights assigned to O&M and LPSP can be adjusted in the objective function; however, the optimal outcome is typically obtained when they are given equal weights. The operating and maintenance cost of the system components during the which can be represented in the following equations:

36 $$\:{\theta\:}_{O\&M}\:=\:{\theta\:}_{PV}+{\theta\:}_{WECS}+{\theta\:}_{FCs}+{\theta\:}_{Batt}+{\theta\:}_{Sc}+{\theta\:}_{ele}$$

Where,

37 $$\:{\theta\:}_{PV}\:=\:\sum\:_{t=1}^{T}{P}_{PV}\left(t\right).{\varnothing\:}_{PV}$$

38 $$\:{\theta\:}_{wind}\:=\:\sum\:_{t=1}^{T}{P}_{W}\left(t\right).{\varnothing\:}_{WECS}$$

39 $$\:{\theta\:}_{FCs}\:=\:\sum\:_{t=1}^{T}{P}_{FCs}\left(t\right).{\varnothing\:}_{FCs}$$

40 $$\:{\theta\:}_{BESS}\:=\:\sum\:_{t=1}^{T}{P}_{batt}\left(t\right).{\varnothing\:}_{BESS}$$

41 $$\:{\theta\:}_{SC}\:=\:\sum\:_{t=1}^{T}{P}_{EV}\left(t\right).{\varnothing\:}_{EV}$$

42 $$\:{\theta\:}_{ele}\:=\:\sum\:_{t=1}^{T}{P}_{ele}\left(t\right).{\varnothing\:}_{ele}$$

RESs exhibit a random and irregular power production nature. Therefore, it is crucial to assess the reliability of a standalone microgrid. The reliability of a microgrid can be evaluated using the concept of LPSP. LPSP represents the probability that the power generation system fails to meet the load requirements during a given period. The calculation for LPSP is as follows^3^:

43 $$\:LPSP=\:\frac{\sum\:_{t=1}^{T}LPS\:\left(t\right)}{{\sum\:_{t=1}^{T}P}_{L}\left(t\right)}\:$$

44 $$\:LPS\left(t\right)={P}_{L}\left(t\right)+{P}_{ele}\left(t\right)-\:{P}_{PV}\left(t\right)-\:{P}_{W}\left(t\right)\:-\:{P}_{FC}\left(t\right)\pm\:\:{P}_{batt}\:\left(t\right)\pm\:{P}_{Sc}\left(t\right)\:$$

### System constraints

#### System reliability constraints

LPSP is a widely used technical indicator to assess the reliability of power systems. It is recommended that the value of LPSP remains below a specified reliability tolerance limit. Mathematically, it can be expressed as:

45 $$\:LPSP\:\le\:\:\epsilon\:$$

#### Operational constraints of BESS

The reliability and longevity of the microgrid heavily depend on a robust and dependable BESS. To maximize the lifespan of the system, it is crucial to avoid overcharging and deep discharging of the BESS. The SOC constraints for the BESS in this study are defined by the following equation. This manuscript focuses on the commonly used SOC range of 80%^52–55^.

46 $$20\% {\text{ }} \le {\text{ }}SOC_{{batt}} \le {\text{ }}80\%$$

#### Operation constraints of SESS

To safeguard the Super capacitor during its operational period, certain constraints have been incorporated. These constraints focus on maintaining the SOC within predefined limits and controlling the charging and discharging rates. These measures are implemented to ensure the longevity and proper functioning of the Super capacitor, promoting stable and controlled operation. The constraints play a crucial role in preventing the Super capacitor from operating beyond its specified capacity and rate limitations, thereby optimizing its performance and extending its operational life.

47 $$50\% {\text{ }} \le {\text{ }}SOC_{{sc}} \le {\text{ }}90\%$$

#### Operation constraints of hydrogen tank

Hydrogen tank capacity must be taken in consideration to ensure safe operation of hydrogen energy storage system this can be presented in the following equation:

48 $$\:{M}_{H2,min}\le\:{M}_{H2}\le\:{M}_{H2,max}$$

#### Power production constraints

To ensure high efficiency and reliability of the proposed system, the power captured from the primary sources, wind and PV, was maximized using two metaheuristic algorithms, namely the AGTO and the ZOA, in conjunction with an ANFIS for an efficient MPPT system. While the power produced and delivered by the different storage systems are limited according to the nominal power of the utilized energy storage components. These constraints can be represented in the following equations:

49 $$\:{P}_{G}\left(t\right)={P}_{w,\:mp}\left(t\right)+{P}_{pv,mp}\left(t\right)$$

50 $$\:{P}_{FC,min}\le\:{P}_{FC}\left(t\right)\le\:{P}_{FC,max}$$

51 $$\:{P}_{sc,min}\le\:{P}_{sc}\left(t\right)\le\:{P}_{sc,max}$$

52 $$\:{P}_{batt,min}\le\:{P}_{batt}\left(t\right)\le\:{P}_{batt,max}\:$$

53 $$\:{P}_{ele,min}\le\:{P}_{ele}\left(t\right)\le\:{P}_{ele,max}$$

#### Power balance constraint

Maintaining a balance between power generation and consumption relies on meeting the power balance constraints. The BESS, Supercapacitor and HESS must match the power consumed to achieve equilibrium. When surplus energy from RESs is available, BESS and SESS discharge power as a positive value, while they take on a negative value when charging is required. These constraints regulate the power flow to align with energy requirements for stable and efficient power balance.

54 $$\:{P}_{L}+{P}_{loss}+{P}_{ele}={P}_{G}\:,\:\forall\:\:t$$

55 $$~P_{{PV}} \left( t \right)\, + \,P_{W} \left( t \right)\, + \,P_{{FCs}} \left( t \right)\, + \:k\:x\:P_{{batt}} \left( t \right)\, + \,k\:x\:P_{{sc}} \left( t \right)\, = \,P_{L} \left( t \right) + P_{{ele}} \left( t \right) + P_{{loss}} \left( t \right)$$

56 $$\:\left\{\begin{array}{c}k=1\:\:\:discharge\:\:\:\:\:\:\:\:\:\:\:\:\:\:\:\:\:\:\:\:\:\\\:k=0\:\:charge\:stays\:constant\:\:\:\:\\\:k=-1\:\:\:charge\:\:\:\:\:\:\:\:\:\:\:\:\:\:\:\:\:\:\:\:\:\:\:\:\end{array}\right.$$

The primary goal of the proposed operational strategy for the backup system, which includes battery banks, fuel cells, and SCs, is to ensure a reliable, cost-effective, and environmentally sustainable energy supply within the microgrid. This approach aims to achieve the following main objectives:

- Reliable Power Supply Assurance: The primary objective of the optimal operation strategy is to ensure a reliable and uninterrupted power supply to critical loads within the microgrid. This involves the strategic deployment of various energy storage technologies, including battery banks, fuel cells, and SCs. By leveraging the unique characteristics of each storage system, the strategy aims to provide immediate responses to short-term disruptions and sustained backup power during extended outages. Through careful coordination and prioritization of these technologies, the system ensures that critical loads receive continuous and reliable power, minimizing the impact of any unforeseen interruptions.
- Efficient Energy Storage Utilization: To achieve efficiency and maximize the lifespan of energy storage technologies, the optimal operation strategy focuses on intelligent energy dispatch. This involves considering the specific capabilities of each storage component—such as the rapid discharge capabilities of SCs and the sustained energy delivery from batteries and fuel cells. By dynamically optimizing the use of these technologies based on real-time load requirements and the availability of RESs, the strategy aims to reduce maintenance costs, extend equipment life, and enhance the overall performance of the microgrid’s backup system.
- Cost-Effective Operation: The strategy places a strong emphasis on cost-effectiveness by minimizing operational expenses and maximizing the economic benefits of integrating renewable energy. Regular cost-benefit analyses guide decision-making, balancing the use of stored energy with external power sources. By optimizing the trade-off between energy storage technologies and external grid power, the strategy seeks to achieve a cost-effective operation that aligns with the microgrid’s financial goals. This includes considering factors such as equipment costs, maintenance expenditures, and potential energy savings in the overall design and operation of the backup system.
- Environmentally Sustainable Practices: Environmental sustainability is a key objective of the optimal operation strategy, emphasizing a reduced reliance on fossil fuels and minimizing greenhouse gas emissions. The strategy prioritizes the deployment of RESs such as solar and wind, integrating them seamlessly with the backup system. By minimizing the environmental impact of the microgrid’s operation, the strategy contributes to broader sustainability goals. It fosters eco-friendly practices by maximizing the use of clean energy and minimizing the carbon footprint associated with the backup system’s operation.
- Adaptability to Dynamic Conditions: The optimal operation strategy is designed to be adaptive, responding effectively to dynamic conditions within the microgrid. This adaptability includes addressing variations in energy demand, fluctuations in renewable energy generation, and unexpected system failures. Real-time monitoring and control mechanisms are integrated into the strategy, enabling the backup system to dynamically adjust its operation. This flexibility ensures that the microgrid can implement load shedding during emergencies, seamlessly integrate with RESs, and maintain a reliable power supply even in the face of changing and unpredictable conditions.

## Results and discussion

### Assessment of the proposed optimizations

**Fig. 7:**
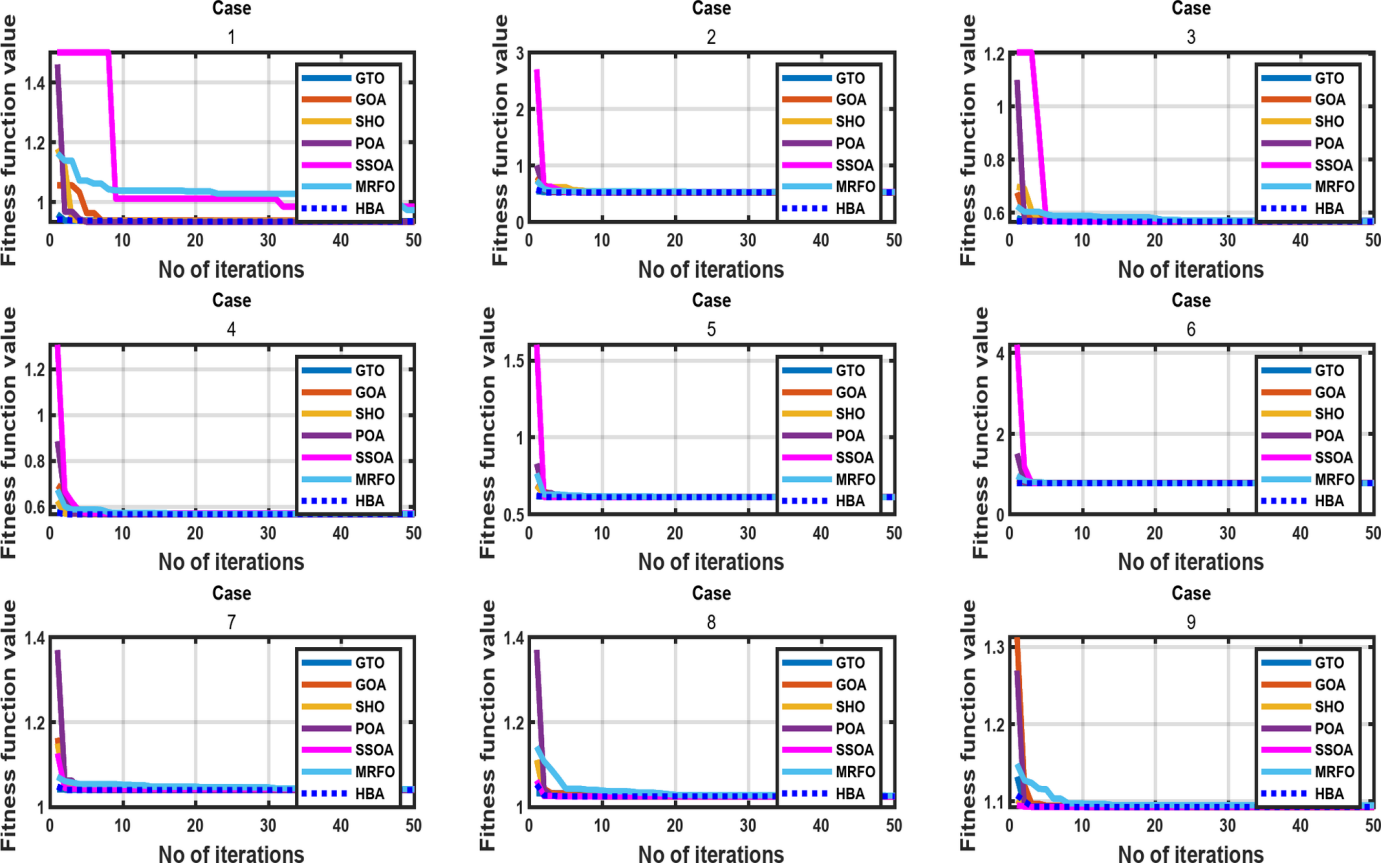
Convergence process across a range of benchmark functions.

This research presents a detailed comparative examination of the performance of seven algorithms: GOA, HBA, MRFO, POA, SHO, SSOA, and GTO. The evaluation encompassed the utilization of nine benchmark test functions, and the convergence processes of each algorithm on these functions are graphically represented in Fig. 7. Table 2 provides an overview of the controlling parameters for each algorithm. The outcomes of the comparative analysis unveiled that HBA exhibited superior performance in terms of both speed and accuracy when reaching optimal solutions. Specifically, HBA displayed a rapid convergence rate towards the optimal solution, requiring fewer iterations to identify the best solution. Additionally, HBA consistently achieved higher levels of accuracy in pinpointing the optimal solution compared to the other algorithms. Its effectiveness in navigating benchmark functions and reaching the optimum solution positions HBA as a highly promising algorithm for tasks demanding a balance of efficiency and accuracy in optimal solution identification.

**Table 2:** Controlling parameters of the proposed algorithms.

Algorithms	Parameters	Value
GOA	Iterations	50
	Population	5000
	PSRs	0.34
	S	0.88
GTO	Iterations	50
	Population	5000
	Beta	1.5
	A	0.4
HBA	Iterations	50
	Population	5000
	Beta	3
	C	3
MRFO	Iterations	50
	Population	5000
	S	2
SHO	Iterations	50
	Population	5000
	U	0.05
	V	0.05
	I	0.05
SSOA	Iterations	50
	Population	5000
	Inertia weight (w)	0.5
	Personal best coefficient (C1)	1
	Global best coefficient (C2)	2
	Inertia weight reduction constant (k)	0.5

### Cases of operation

The examination of the proposed system involved comprehensive evaluations spanning a spectrum of operational scenarios. Three distinct cases were systematically presented, each characterized by varying degrees of involvement of backup sources. These cases were meticulously analyzed to assess the system’s performance under different conditions and to provide a nuanced understanding of its adaptability across a range of operational contexts.

#### 1st scenario

**Fig. 8:**
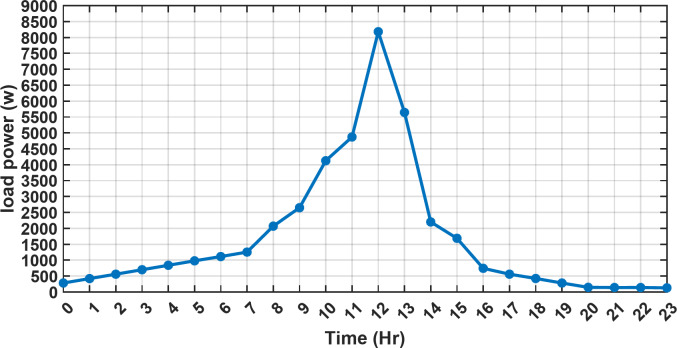
The fluctuations in power consumption over the entire duration of a day.

**Fig. 9:**
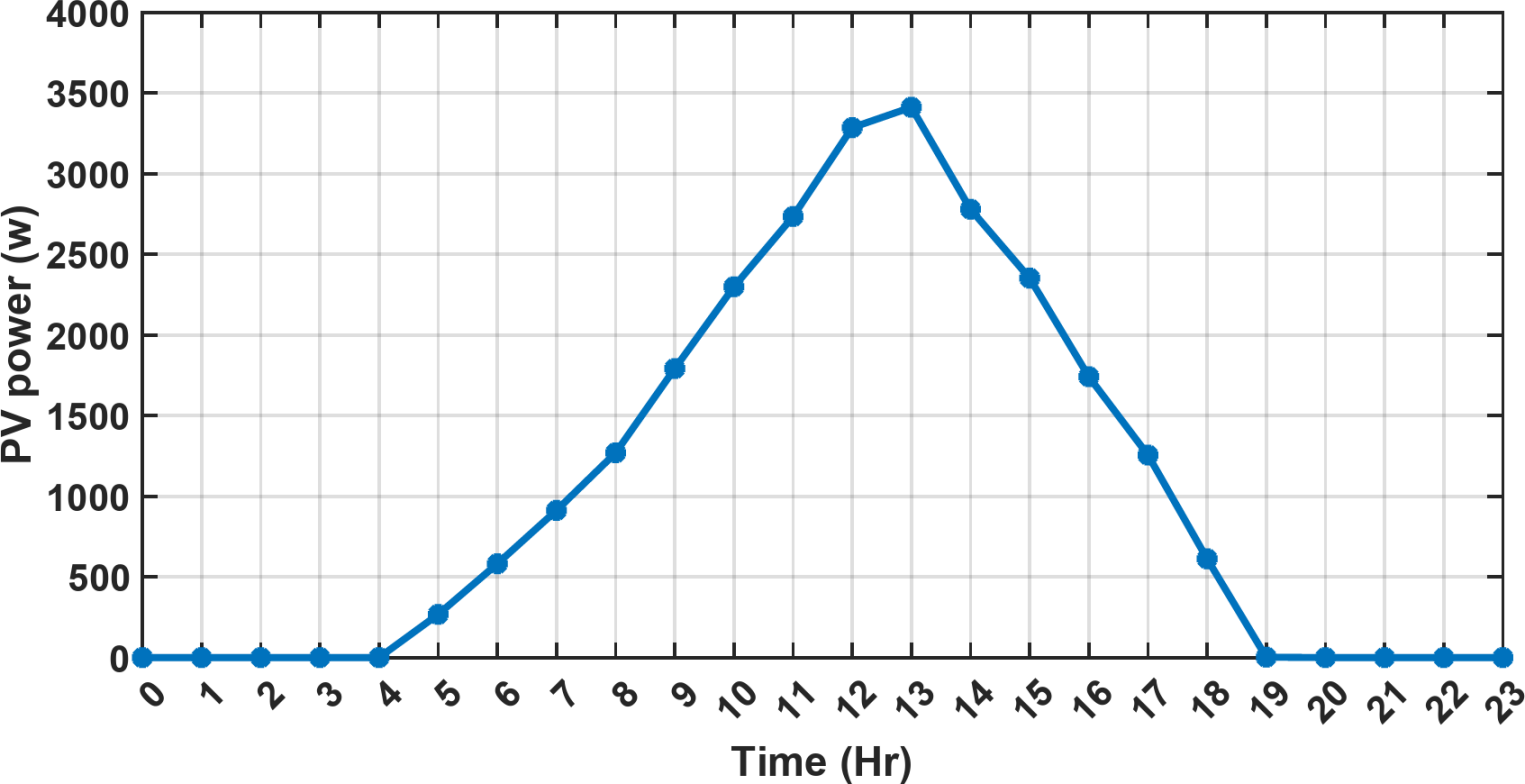
The fluctuations in PV power over the entire duration of a day.

**Fig. 10:**
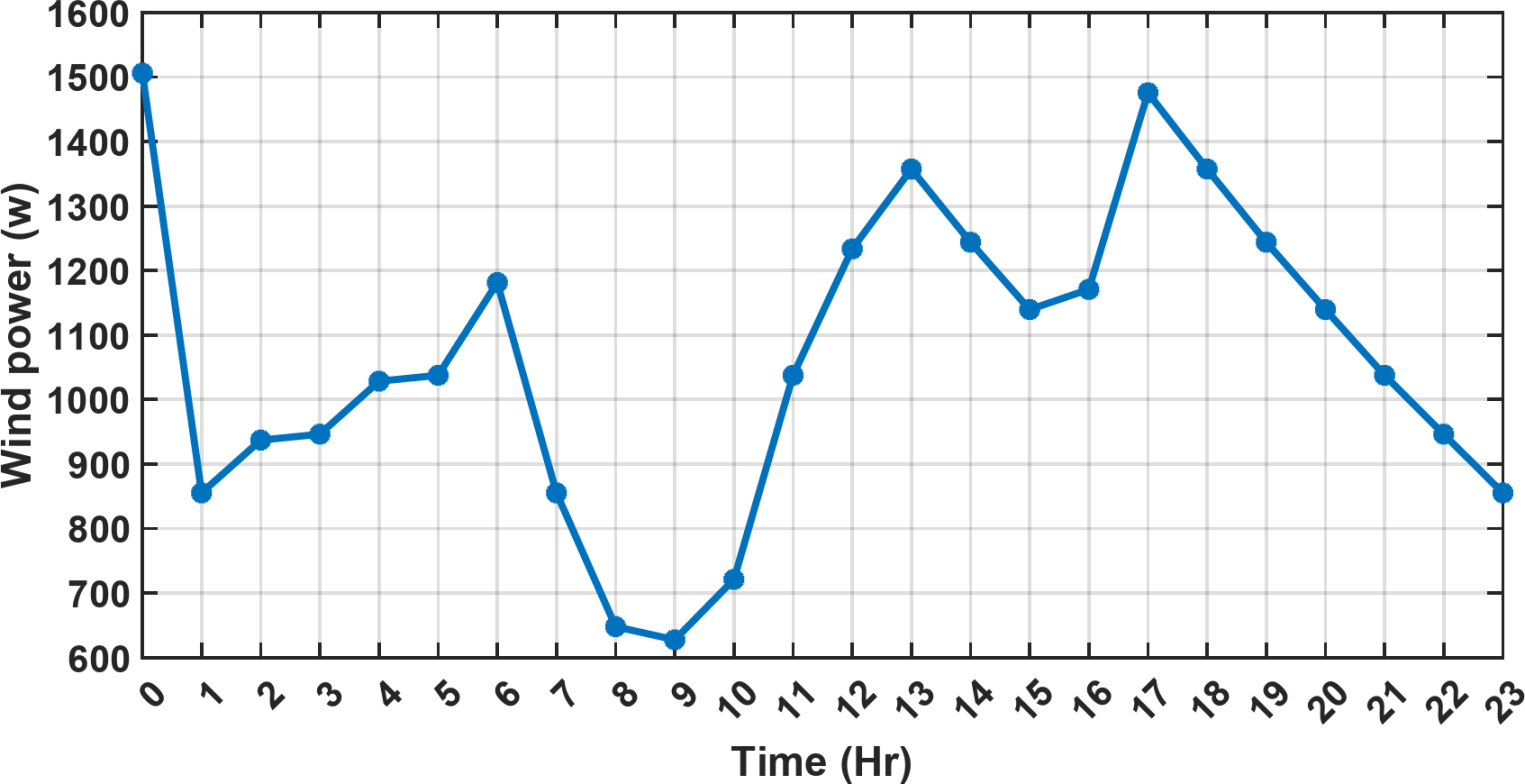
The fluctuations in wind power over the entire duration of a day.

**Fig. 11:**
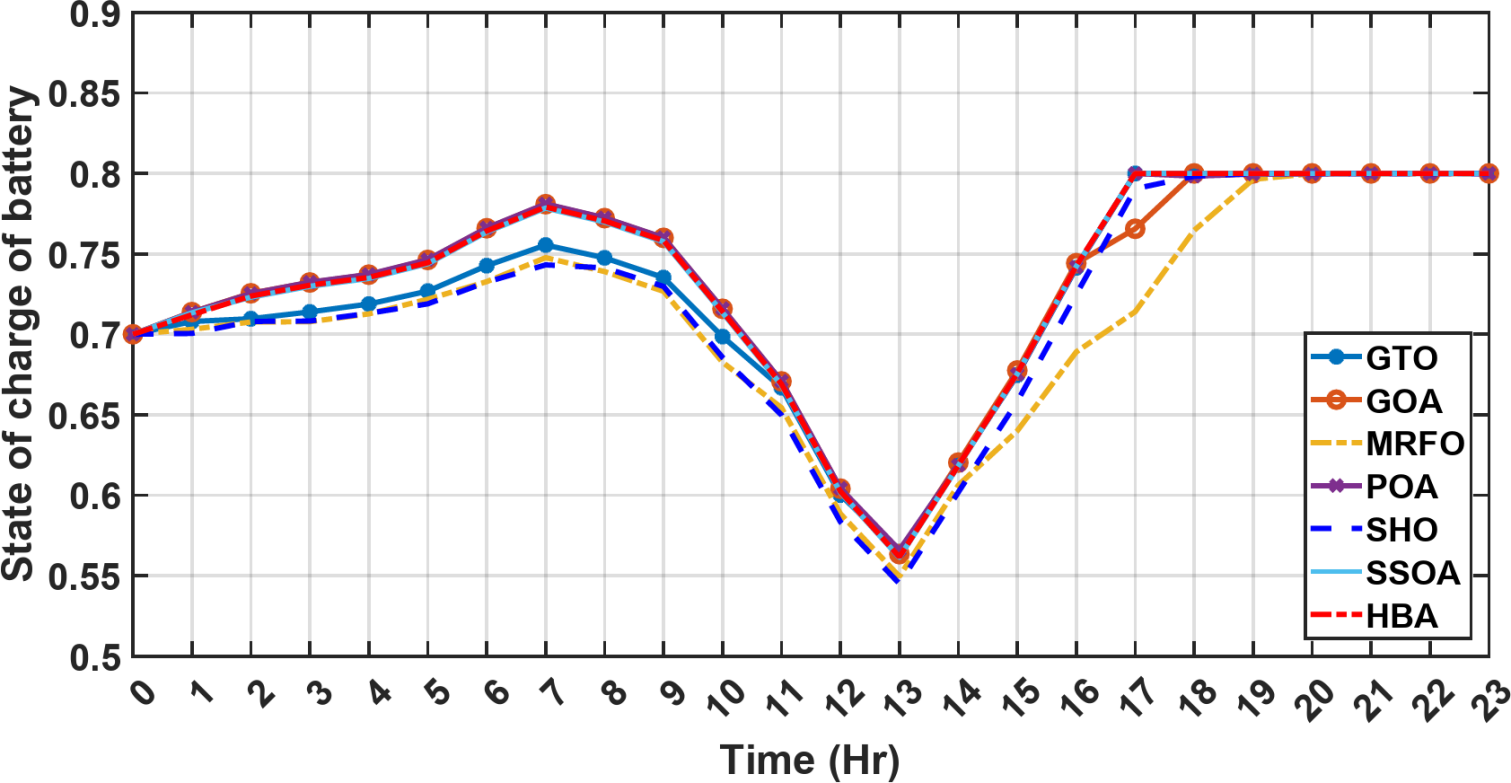
The fluctuations in SOC of the BESS over the entire duration of a day in the first scenario.

**Fig. 12:**
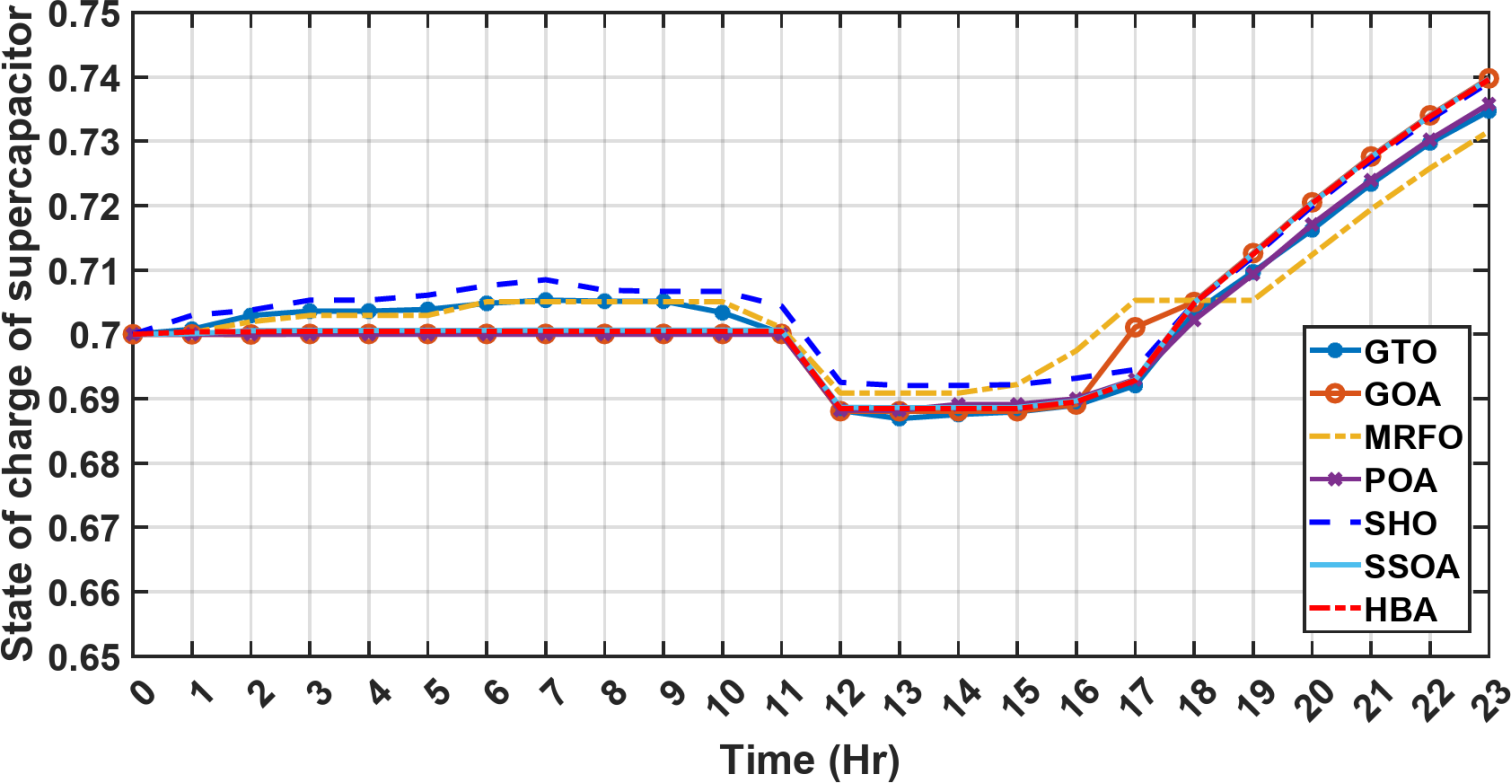
The fluctuations in SOC of the supercapacitor over the entire duration of a day in the first scenario.

In the first operational scenario, the system ensured power demand satisfaction through a dual approach involving both primary and backup energy sources. The battery’s charge was carefully maintained within the 20–80% range, while the supercapacitor retained a charge level spanning from 50 to 90%. Additionally, the hydrogen tank contained an optimal volume ranging from 1 kg to 5 kg, ensuring a consistent supply of hydrogen for the fuel cell’s power generation. Notably, this operational mode operated seamlessly without the need for load shedding or resorting to a dump load. The energy storage system efficiently compensated for any shortfall in power, particularly when primary energy sources alone fell short of meeting the load demand. The fluctuations in power consumption over the entire duration of a day are shown in Fig. 8. Table 3 shows the results for 7 metaheuristic algorithms optimizing energy generation costs over 24-hour periods. The HBA achieved the lowest overall cost of $33.93232, outperforming all other algorithms. In second place was GOA which had a cost of $33.932836. While GOA was a close second, HBA still improved upon its performance by 0.002%. The third best performing algorithm was GTO with a cost of $34.01792. The HBA lowered costs by 0.252% compared to GTO, demonstrating stronger optimization ability. When measured against MRFO cost of $34.21107, HBA provided a 0.744% enhancement. An even greater difference of 2.033% was seen versus POA at $34.62075. Compared to the SHO cost of $34.00911, HBA’s results were 0.237% better. The largest magnitude of outperformance occurred against SSOA, where HBA lowered costs by 6.925% over SSOA’s $36.28691 outcome. In all cases, the HBA exhibited the most effective optimization, minimizing energy generation costs through percentages ranging from 0.002 to 6.925% below the next best alternatives. The HBA algorithm stands out with an impressively low value of 7.9 × 10^− 10^, reflecting its superior performance and exceptional reliability. This minimal value positions HBA as the top-performing algorithm in the given context. Conversely, SSOA exhibits the highest value of 2.78 × 10^− 6^, pointing towards its comparatively poorer performance and lower reliability in Case 1. This elevated value designates SSOA as the least favorable algorithm in terms of both performance and reliability within this scenario. HBA performed the best among the algorithms with an efficiency of 95.893%. POA had the lowest efficiency at 95.563% which was 0.333% lower than HBA, indicating it had the poorest performance in this case. The next highest performing algorithms, GOA, SEA and MRFO, all achieved efficiencies that were within 0.003% of HBA’s top score. Specifically, GOA achieved 95.890%, SEA achieved 95.891% and MRFO achieved 0.95826%, demonstrating that they closely trailed the leading performance of HBA, while still outpacing POA which evidenced the largest difference versus the top algorithm in this first case. HBA had the lowest computation time of 569.3516 s, making it the fastest optimizer by a clear margin. POA took the second lowest time of 993.4645 s, which was 424.1128 s slower than HBA. GOA, MRFO and SEA followed with times of 1058.4, 998.6014 and 835.0582 s respectively, all significantly slower than HBA by between 265 and 488 s. SSOA recorded the second fastest time of 640.2090 s, still over 70 s slower than HBA. The PV power output undergoes noticeable fluctuations throughout the entire day, reflecting the dynamic nature of solar energy generation. The peak of power production is observed around midday, precisely at 1 pm, corresponding to the period when solar irradiation reaches its maximum intensity. This temporal pattern aligns with the high levels of sunlight exposure during this timeframe. Figure 9 demonstrates this peak in power output, highlighting the significant impact of solar irradiation variations on the daily energy generation profile. Wind power exhibits noticeable fluctuations throughout the entire day, indicating the dynamic nature of wind energy production. The pinnacle of power generation is particularly noteworthy, occurring at 5 pm when wind speeds reach their highest levels. This temporal pattern underscores that the wind system achieves its maximum energy output during periods of heightened wind intensity, making a substantial contribution to the overall daily power profile. The detailed representation revealed in Fig. 10 clarifies how wind power plays a crucial role in shaping the overall energy pattern throughout the day. The electrical load reached its peak, necessitating the activation of backup sources to address the increased demand. The SOC for both the BESS and SCs are represented in Figs. 10, 11. Around 1 pm, a noticeable decline in SOC is observed in both BESS and SCs. This decline indicates a period of increased energy consumption or a higher demand than the instantaneous generation capacity. However, the response strategies of the two storage systems differ. Following this decline, there was a distinctive divergence in the charging patterns. The SOC of the BESS started to ascend after 1 pm, steadily increasing until it reached its maximum at 5 pm. This indicates a successful replenishment of stored energy during the post-peak period. Conversely, the supercapacitor’s SOC exhibited a different trend, showing an ascent after 5 pm as depicted in Fig. 12. This suggests a delayed but gradual accumulation of energy within the supercapacitor system.

**Table 3:** Daily operational costs of seven algorithms in the 1st scenario.

Time (hr)	Operating costs ($)						
	GOA	GTO	HBA	MRFO	POA	SHO	SSOA
1	0.933167557	0.934889	0.93305	0.965719	0.932819	0.933903	0.983776
2	1.453313129	1.45847	1.452813	1.490187	1.452529	1.453717	1.514957
3	2.018974949	2.027672	2.018654	2.057992	2.018191	2.019897	2.082543
4	2.585060867	2.594443	2.584741	2.626279	2.584196	2.58597	2.652925
5	3.197185588	3.206614	3.196856	3.238448	3.196312	3.198085	3.265044
6	3.967179798	3.976909	3.966842	4.009447	3.966296	3.968068	4.035046
7	5.006862878	5.017663	5.006528	5.051218	5.005979	5.007752	5.077145
8	6.031690865	6.043064	6.031355	6.077882	6.030805	6.032598	6.101967
9	7.124278859	7.13584	7.123941	7.172336	7.123391	7.12519	7.194552
10	8.497196972	8.508761	8.496856	8.545534	8.496306	8.498109	8.567464
11	10.24014265	10.25373	10.23981	10.28879	10.23925	10.24106	10.31041
12	12.4115117	12.42876	12.41117	12.46292	12.41062	12.41242	12.4864
13	18.97147575	19.03905	18.97113	19.25306	19.65452	19.04819	21.30342
14	21.70058008	21.76937	21.70025	21.98248	22.38094	21.77691	24.03054
15	24.03152119	24.10103	24.0312	24.31351	24.71231	24.10785	26.36124
16	26.06537221	26.13536	26.06505	26.34779	26.74543	26.1417	28.41793
17	27.79660861	27.86656	27.79628	28.07951	28.4757	27.87294	30.15384
18	29.44258474	29.51224	29.45216	29.72334	30.12056	29.51883	31.82114
19	30.65168078	30.72389	30.65119	30.92983	31.34429	30.72797	33.01547
20	31.43036839	31.50746	31.42986	31.70819	32.12168	31.50665	33.78549
21	32.14774887	32.2298	32.14724	32.42564	32.8382	32.22403	34.50203
22	32.80059096	32.88265	32.80008	33.0786	33.49001	32.87687	35.1547
23	33.39538025	33.47742	33.39487	33.67348	34.08426	33.47166	35.74946
24	33.93283636	34.01792	33.93232	34.21107	34.62075	34.00911	36.28691

**Fig. 13:**
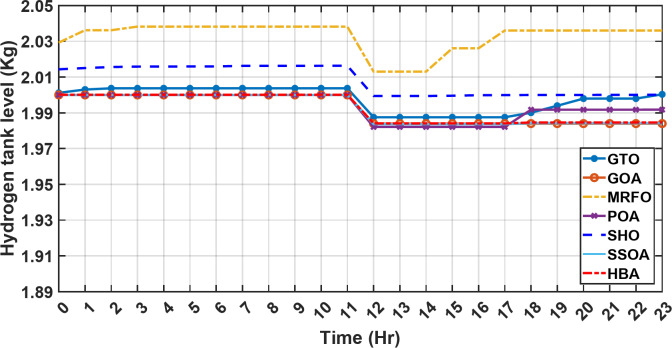
The fluctuations in the hydrogen tank level over the entire duration of a day in the first scenario.

**Fig. 14:**
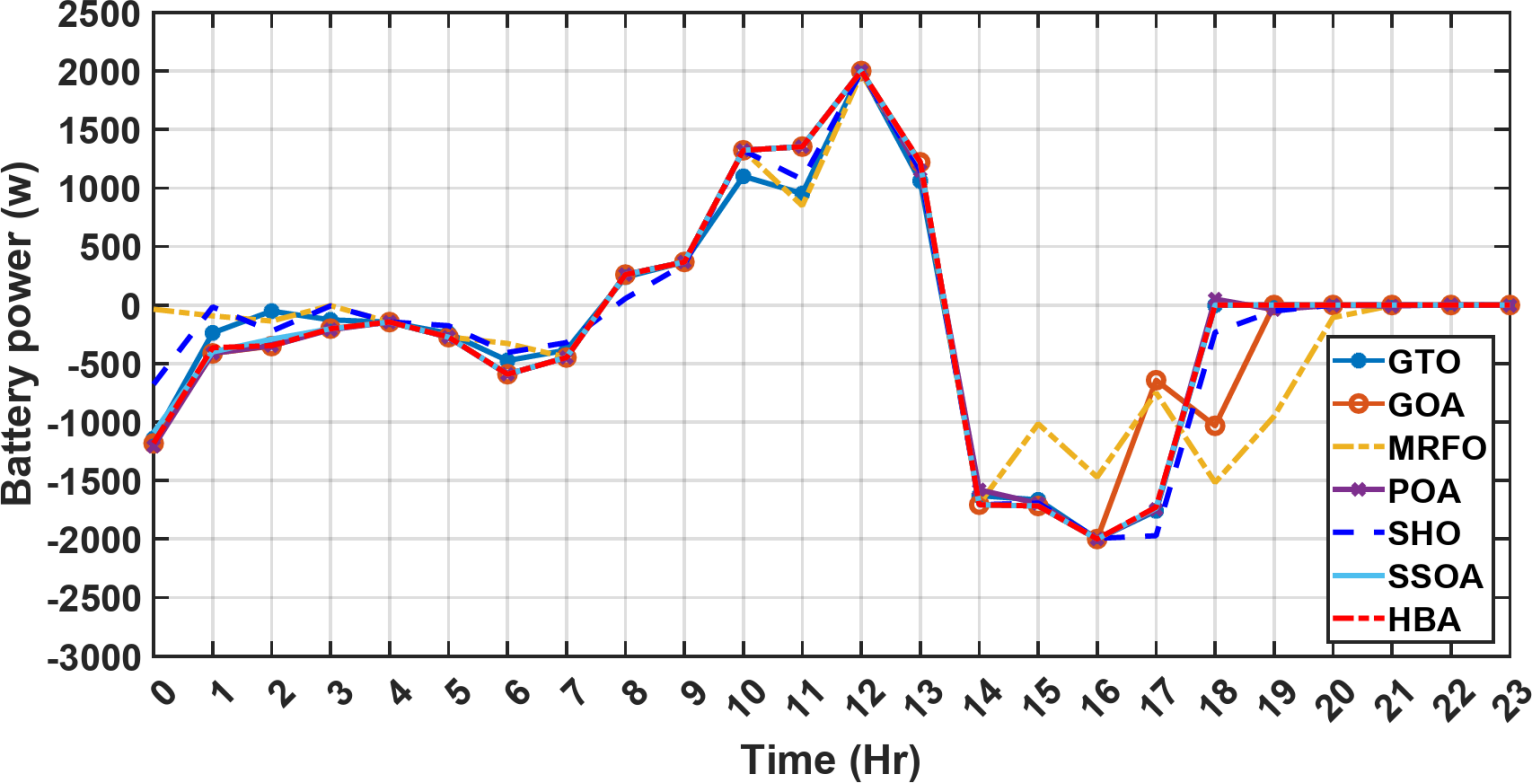
The fluctuations in the power of the BESS over the entire duration of a day in the first scenario.

**Fig. 15:**
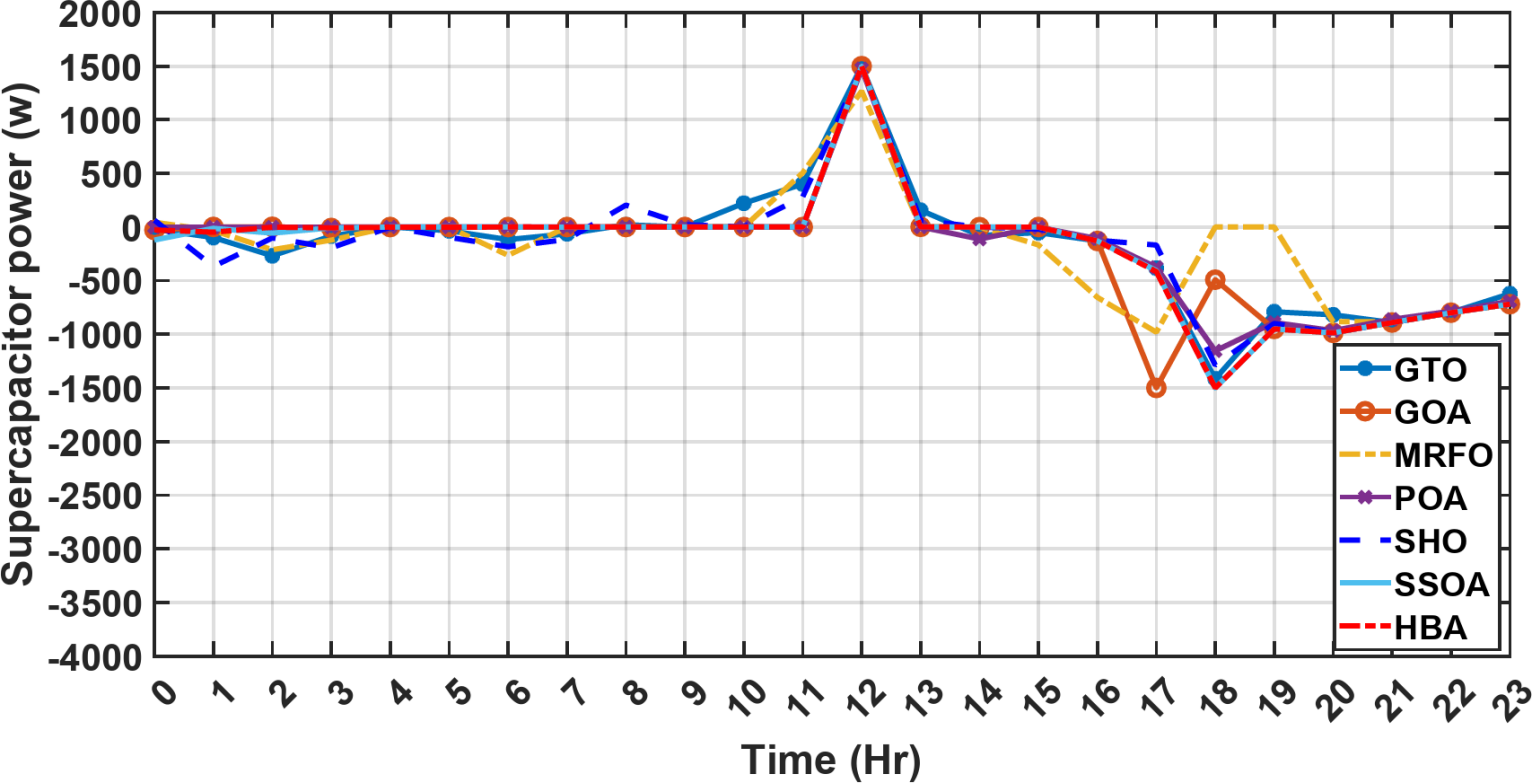
The fluctuations in the power of the supercapacitor over the entire duration of a day in the first scenario.

**Fig. 16:**
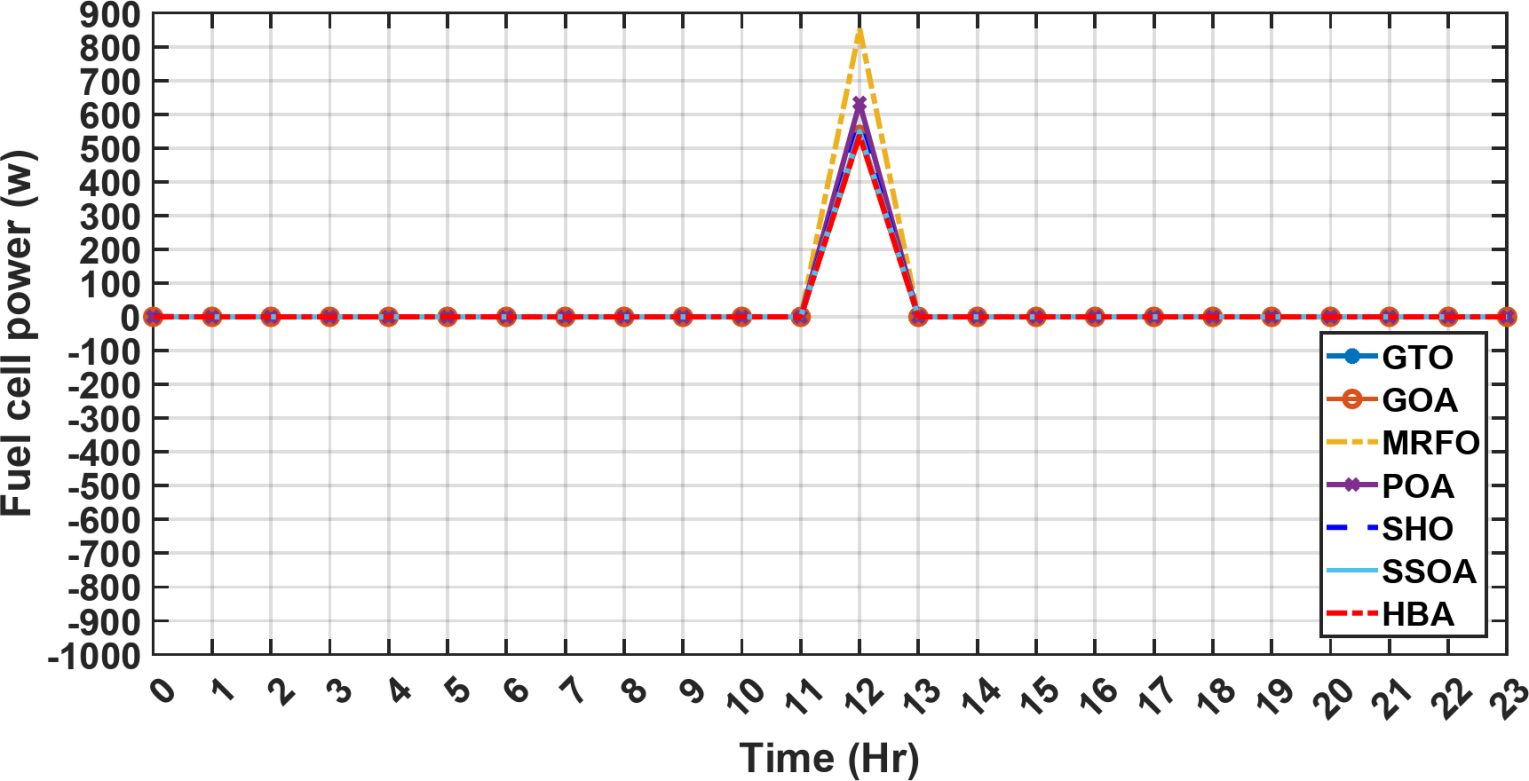
The fluctuations in the power of fuel cell over the entire duration of a day in the first scenario.

Figure 13 illustrates the hydrogen tank level, demonstrating a decrease from 12 pm to 6 pm. This decline in hydrogen tank level corresponds to a period of heightened demand or reduced availability of primary energy sources. Notably, the backup sources, comprising both the BESS and SCs, played a significant role in power contribution during this period. The power contributed by the BESS is detailed in Fig. 14, while Fig. 15 provides a representation of the supercapacitor’s power contribution. The backup sources’ notable power contribution is indicative of their crucial role in bridging energy gaps and ensuring a stable power supply during periods of increased demand or fluctuations in primary energy generation. Moreover, Fig. 16 elucidates the power generated by fuel cell, shedding light on the hydrogen production process during the specified timeframe. These figures collectively offer valuable insights into the dynamic interplay of various energy storage and generation components within the system.

#### 2nd scenario

**Fig. 17:**
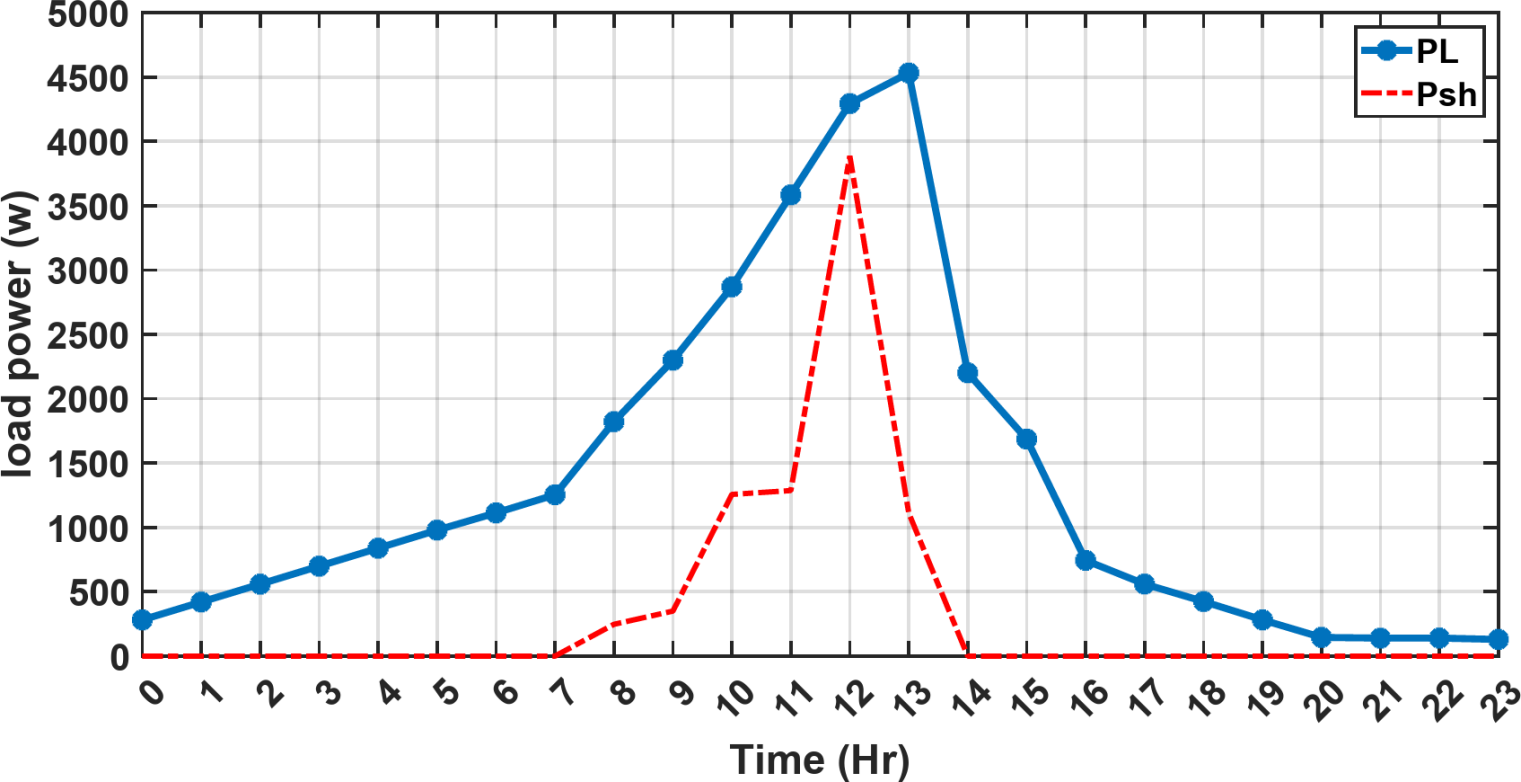
The variations in both power consumption and instances of load shedding throughout the entire day in the second scenario.

**Fig. 18:**
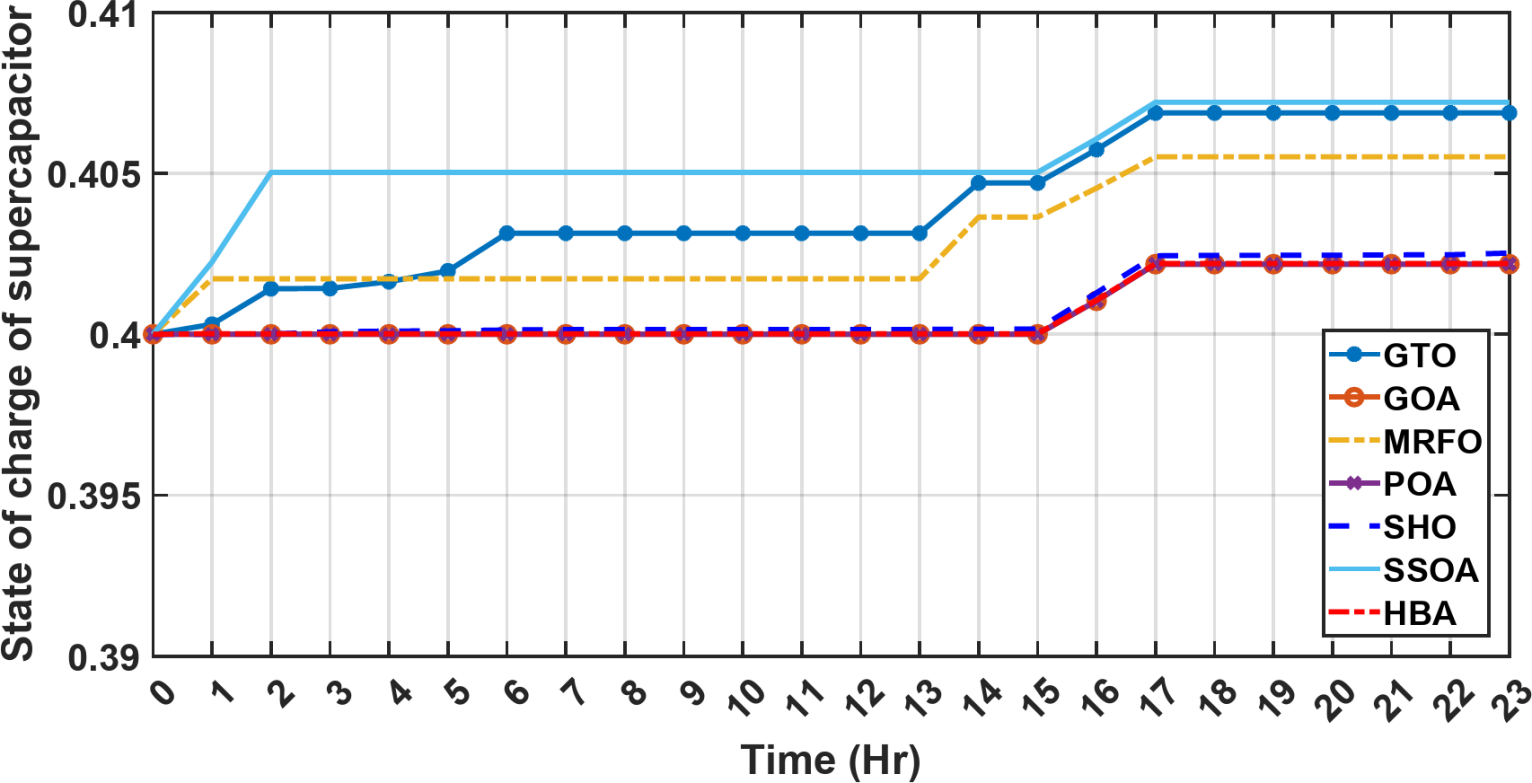
The fluctuations in SOC of the supercapacitor over the entire duration of a day in the second scenario.

**Fig. 19:**
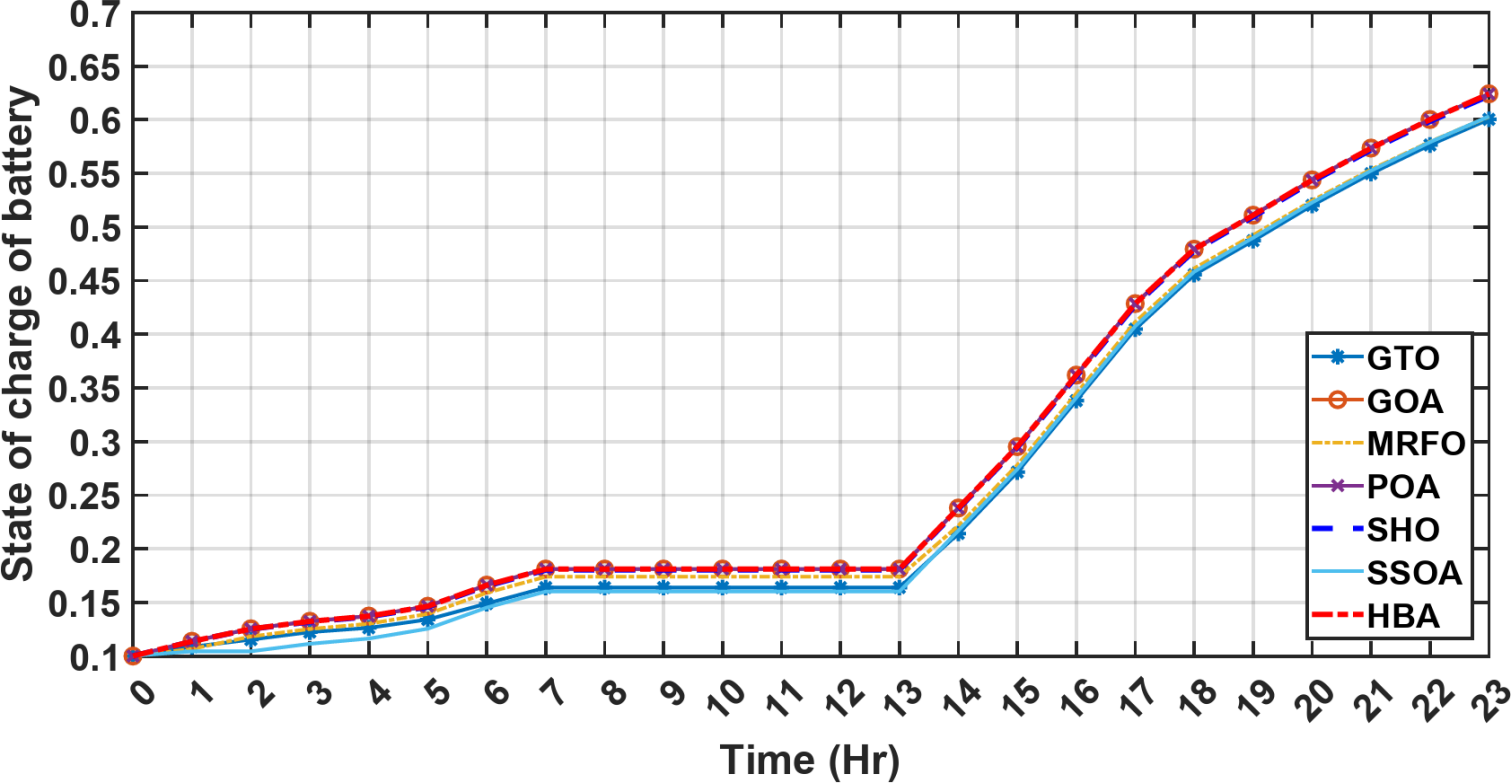
The fluctuations in SOC of the BESS over the entire duration of a day in the second scenario.

In the second operational scenario, where power demand exceeded the generated capacity due to charge limitations on all energy storage systems, the proposed EMS demonstrated remarkable adaptability. It swiftly executed accurate load-shedding decisions, strategically optimizing power distribution and thereby ensuring sustained operational stability. Notably, in this specific scenario, the backup sources, including both fuel cells and the supercapacitor, were intentionally taken out of service. This deliberate action was implemented to assess the system’s capability to handle a situation where an increased load surpasses the energy generation capacity. The objective was to gauge the system’s resilience and effectiveness in managing the energy demand when both backup and primary sources are unable to cover the required energy demand. This deliberate scenario sheds light on the EMS’s ability to make informed decisions under challenging conditions, emphasizing its role in maintaining operational stability and managing energy constraints effectively. It serves as a valuable test of the microgrid system’s robustness and adaptability in scenarios where traditional backup sources are unavailable or face limitations. Over the 24-hour period, HBA achieved the lowest overall energy cost of $29.69141785 as shown in Table 4. This was marginally better than SSOA at $29.69407619, with HBA lowering costs by 0.0004%. GOA had the third best overall performance at $29.6914613, but HBA still reduced costs by 0.00001745%, demonstrating its stronger cost optimization abilities. When measured against GTO’s cost of $29.71705, HBA decreased expenses by 0.00793215%, a greater savings. MRFO incurred $29.69305 in costs, yet HBA cut this by 0.00063135%. An even larger percentage cost reduction of 0.000782395% was seen versus POA’s $29.6914. HBA also enhanced savings compared to SEA’s $29.71596 outcome by 0.00934235%. Therefore, when directly comparing all algorithms over the 24-hour period, HBA consistently found the most cost-effective solution, evident through its ability to minimize energy costs between 0.00001745 and 0.00934235% lower than alternatives like GOA, GTO, MRFO, POA and SEA, clearly exhibiting the most powerful cost optimization capabilities of the metaheuristic approaches evaluated. HBA distinguishes itself as the top performer and exhibits the highest reliability, marked by its lowest value of 0. This indicates superior performance and reliability, positioning HBA as the most favorable algorithm within the given context. Conversely, SSOA records the highest value of 9.56 × 10^− 5^, highlighting its status as the worst performer with the lowest reliability in Case 2. This elevated value designates SSOA as the least preferable algorithm, signifying its comparatively poorer performance and lower reliability in this scenario. HBA and MRFO tied for the top performance among the algorithms with an identical efficiency score of 96.691%. SSOA had the lowest efficiency at 96.320% which was 0.371% lower than the leading pair of HBA and MRFO, indicating it had the poorer result. The remaining algorithms, GTO, POA and SEA, achieved efficiencies that were all within 0.006% of the top score tied by HBA and MRFO. Specifically, GTO achieved 96.650%, POA achieved 96.684% and SEA achieved 96.656%, demonstrating performances closely clustered around the joint leading scorer, while SSOA showed the greatest variance versus the top performing algorithms in this second case test. HBA once again demonstrated the best performance with a computation time of 572.4453 s. POA and MRFO followed with times of 982.2214 and 1009.3 s respectively, both over 400 s slower than HBA. SSOA and SEA recorded the next fastest times of 617.4670 and 917.0254 s respectively, while GOA was the slowest of the test algorithms at 1024.5 s, over 450 s slower than HBA. In the second scenario, the fluctuations in both power consumption and instances of load shedding throughout the entire day are presented in Fig. 17. Both PV and wind power outputs exhibit noticeable variations, with the peak of power production occurring around midday. The dynamic nature of wind energy production is evident through the significant fluctuations observed throughout the day. The electrical load experienced its peak, prompting the activation of backup sources to address the heightened demand and instances of load shedding. As was previously explained, all reserve sources cannot cover the deficit in this scenario. As previously mentioned, it’s crucial to note that all reserve sources are unable to cover the deficit in this scenario, highlighting the system’s limitations under specific conditions. Figures 18, 19 illustrate the SOC for both the BESS and SCs. At the beginning of the day, the supercapacitor’s SOC was 0.4, reaching 0.4056 by the end of the day. In contrast, the BESS started with an SOC of 0.1 and reached 0.62 by the day’s end, indicating a distinctive divergence in the charging patterns between the two energy storage systems. This distinct divergence in charging patterns signifies the different charging behaviors and utilization strategies between the supercapacitor and BESS components. These figures provide a comprehensive view of the energy dynamics in the second scenario, encompassing power generation, load shedding, and the unique charging behaviors of energy storage systems.

**Table 4:** Daily operational costs of seven algorithms in the 2nd scenario.

Time (hr)	Operating costs ($)						
	GOA	GTO	HBA	MRFO	POA	SHO	SSOA
1	0.932797908	0.94819	0.932797904	0.93344	0.932798	0.951711	0.93693279
2	1.452545737	1.47269	1.452503804	1.453365	1.452504	1.473981	1.458608724
3	2.018197507	2.04003	2.018155573	2.019089	2.018156	2.042818	2.024283608
4	2.584201279	2.606044	2.584159343	2.5852	2.584159	2.608822	2.590277802
5	3.196315821	3.218396	3.196273868	3.197352	3.196274	3.220936	3.202399279
6	3.966298829	3.988762	3.966256877	3.967347	3.966257	3.990919	3.972386194
7	5.005981837	5.029781	5.005939768	5.007112	5.005944	5.030607	5.012050155
8	6.030807147	6.054607	6.030765085	6.031962	6.030768	6.055432	6.036894924
9	7.113894439	7.137695	7.113852377	7.11505	7.113857	7.13852	7.119982216
10	8.473359312	8.497159	8.47331725	8.474515	8.473321	8.497984	8.479447089
11	10.16799192	10.19179	10.16794986	10.16915	10.16795	10.19262	10.1740797
12	12.28989592	12.3137	12.28985386	12.29105	12.28986	12.31452	12.2959837
13	14.83150342	14.8553	14.83146136	14.83266	14.83147	14.85613	14.8375912
14	17.51595522	17.53976	17.51591316	17.51711	17.51592	17.54058	17.522043
15	19.84689411	19.87246	19.84685207	19.84806	19.84675	19.87141	19.856618
16	21.88074176	21.90633	21.88069971	21.88192	21.8806	21.90525	21.88969777
17	23.61197793	23.63756	23.61193588	23.6133	23.61182	23.63649	23.62014049
18	25.25545922	25.28105	25.25541717	25.25683	25.25531	25.27996	25.26267363
19	26.45000475	26.4756	26.44996163	26.45143	26.44984	26.47451	26.45645498
20	27.21999984	27.24559	27.21995675	27.22146	27.21984	27.2445	27.22568189
21	27.92837171	27.95397	27.92832847	27.92986	27.92821	27.95287	27.93328667
22	28.57308205	28.59868	28.57303863	28.57459	28.57292	28.59758	28.57722912
23	29.16057136	29.18616	29.16052793	29.16209	29.16041	29.18507	29.16395367
24	29.6914613	29.71705	29.69141785	29.69305	29.6914	29.71596	29.69407619

**Fig. 20:**
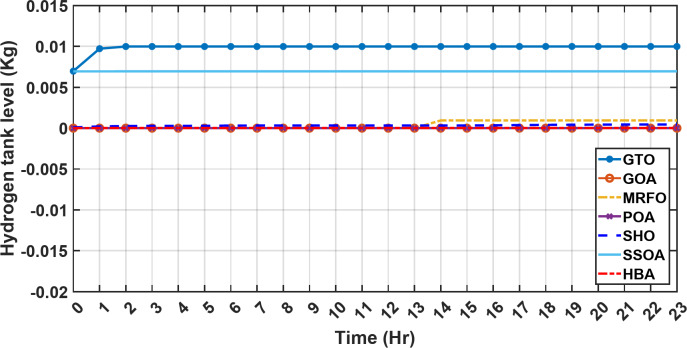
The fluctuations in the hydrogen tank level over the entire duration of a day in the second scenario.

**Fig. 21:**
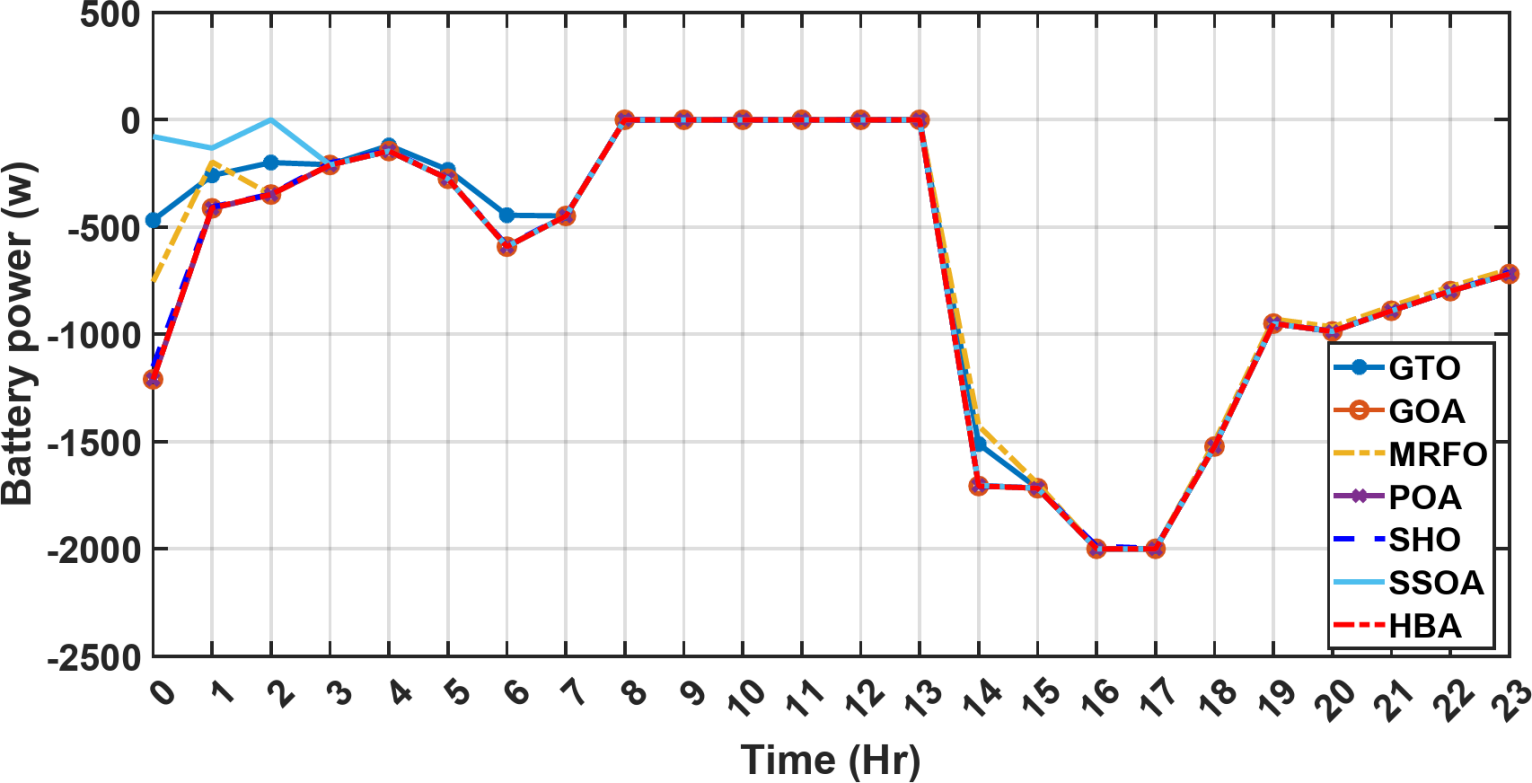
The fluctuations in the power of the BESS over the entire duration of a day in the second scenario.

**Fig. 22:**
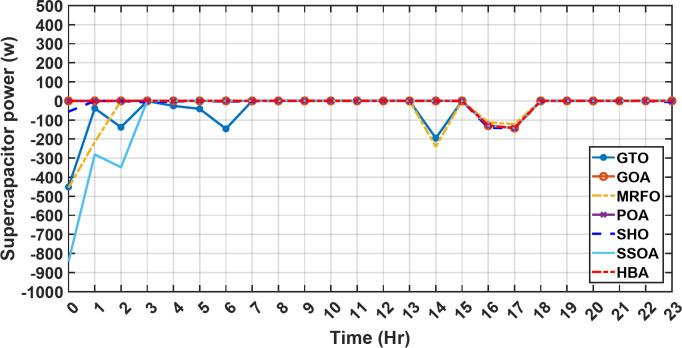
The fluctuations in the power of the supercapacitor over the entire duration of a day in the second scenario.

**Fig. 23:**
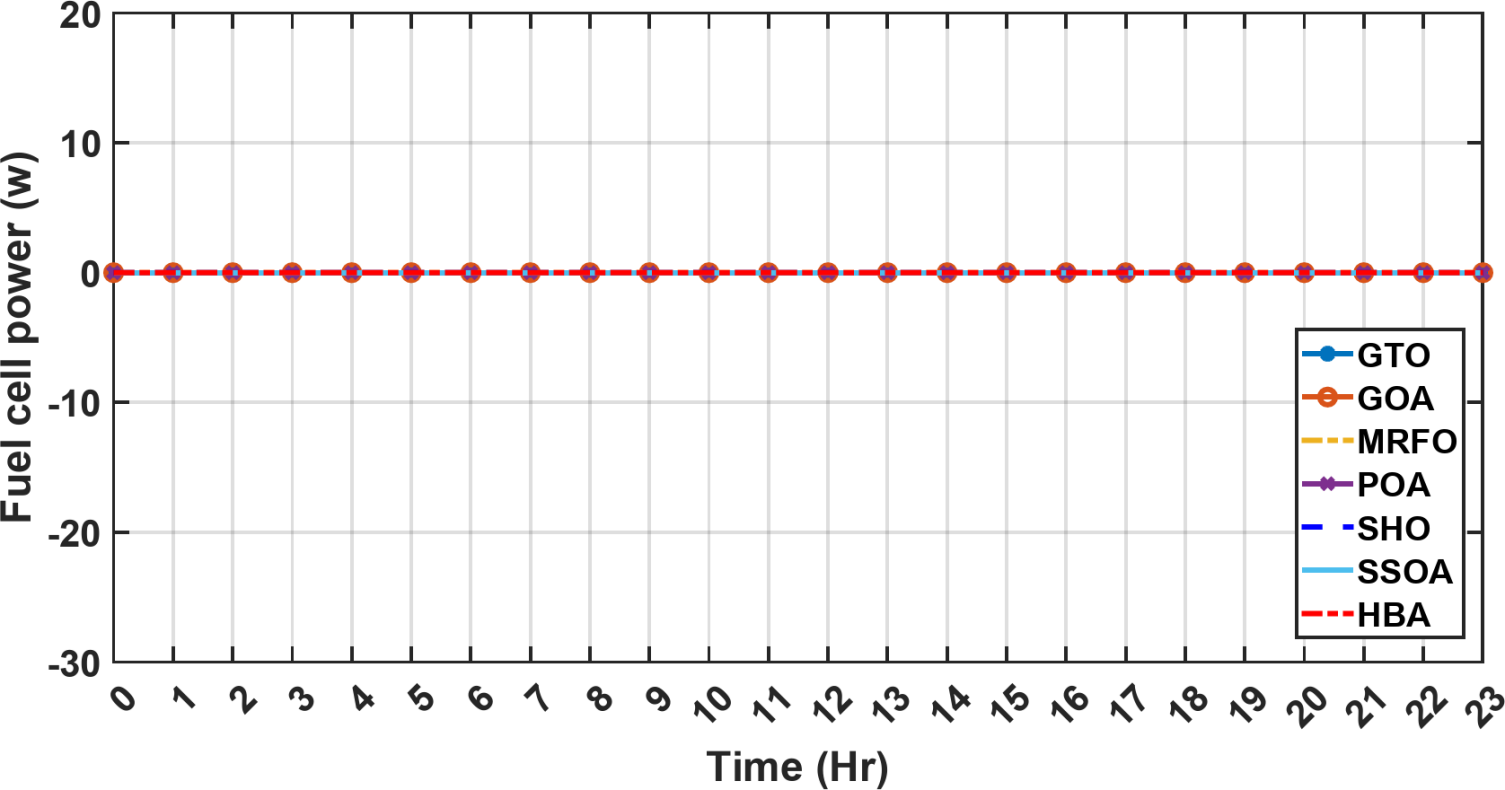
The fluctuations in the power of fuel cell over the entire duration of a day in the second scenario.

In Fig. 20, the hydrogen tank level is illustrated, revealing a near-zero level during the depicted period. This circumstance highlights the challenge of relying on backup sources, including both the BESS and SCs. The specific power contributions of the BESS are delineated in Fig. 21, while Fig. 22 provides a representation of the power contributed by the supercapacitor. Additionally, it is crucial to emphasize that, in this specific scenario, Fig. 23 indicates that fuel cell did not generate any power due to the lack of hydrogen in the hydrogen tank. The dynamics of these backup sources play a pivotal role in ensuring the reliability and resilience of the energy system. The system adeptly resorted to load shedding as a mitigation strategy due to the incapacity of reserve resources to compensate for the deficit. This scenario effectively demonstrated the system’s capability to navigate situations in which both reserve and primary sources prove insufficient to meet the energy demand. It underscores the importance of robust load management strategies and showcases the system’s adaptability in handling challenging conditions to maintain operational stability.

#### 3rd scenario

**Fig. 24:**
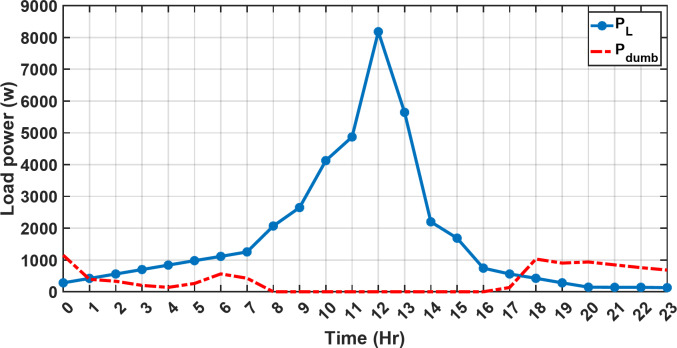
Fluctuations in both power consumption and occurrences of dump power throughout the entire day in the third scenario.

**Fig. 25:**
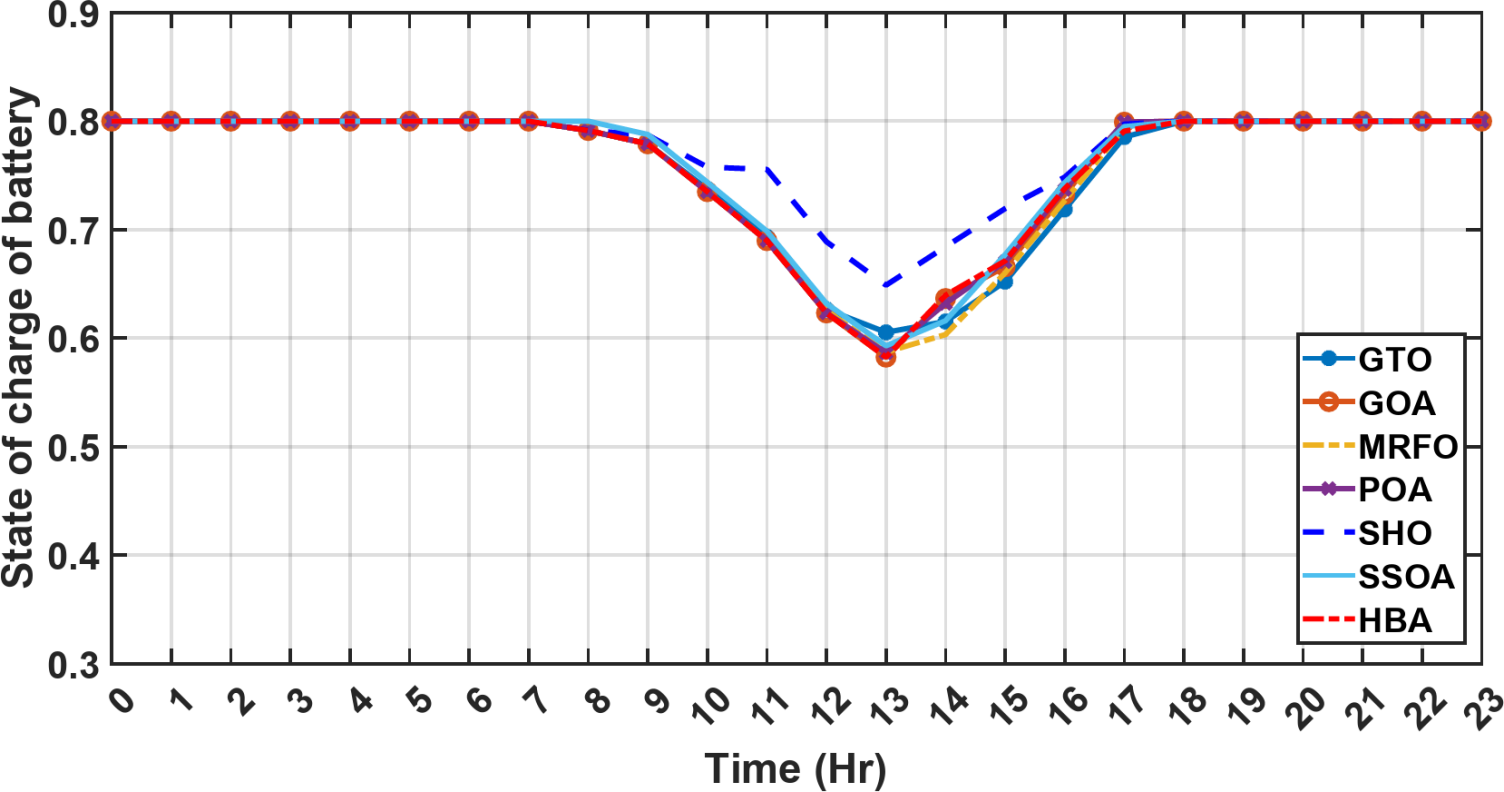
The fluctuations in SOC of the BESS over the entire duration of a day in the third scenario.

**Fig. 26:**
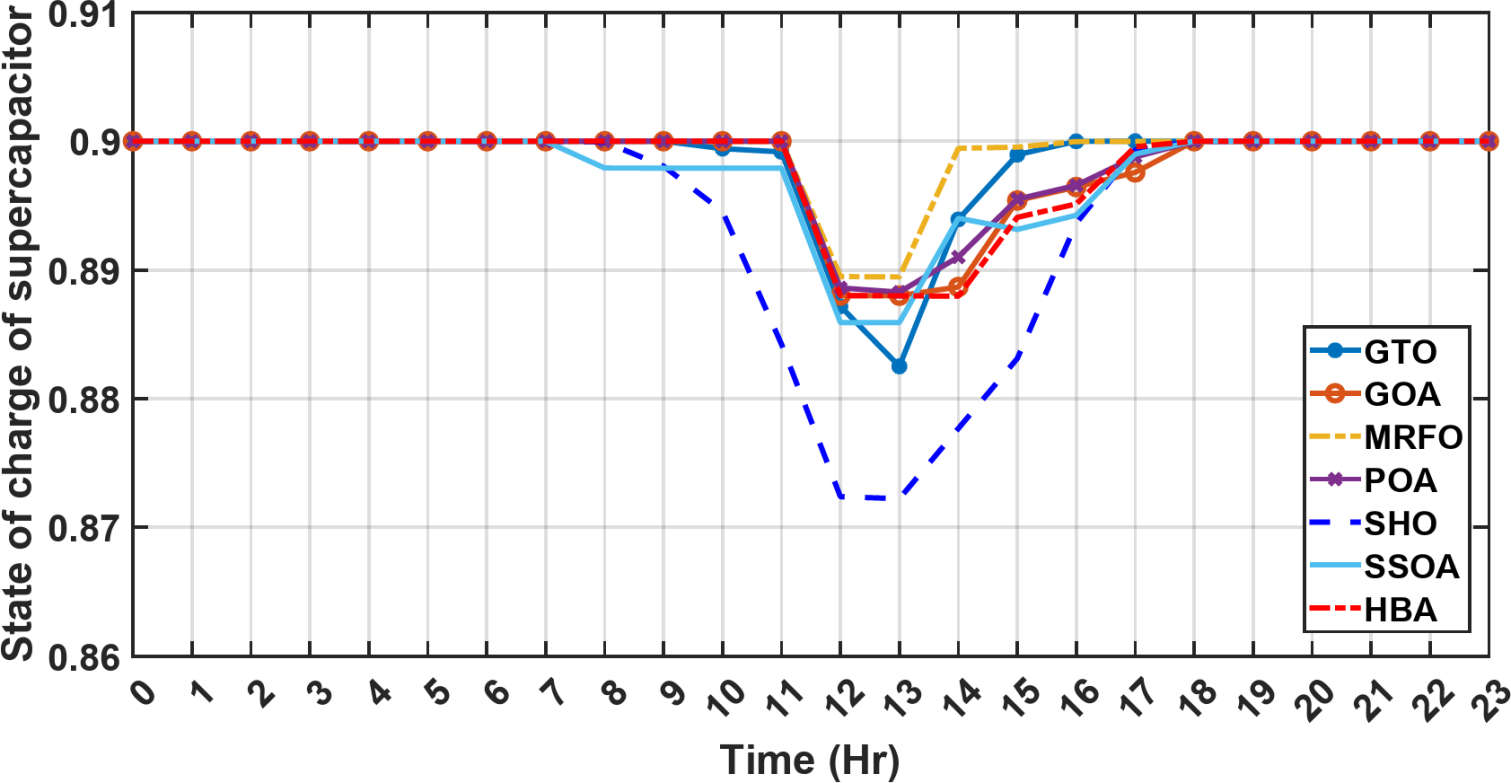
The fluctuations in SOC of the supercapacitor over the entire duration of a day in the third scenario.

In the third operational scenario, where the generated power exceeded the immediate load demand and the storage system components reached full capacity, the proposed EMS adeptly rerouted surplus power to auxiliary loads (dump loads). This strategic redirection prevented the overcharging of storage elements and enhanced grid stability. This innovative functionality not only optimized energy utilization but also significantly contributed to the overall resilience and sustainability of microgrid operations. In this scenario, the HBA has the lowest average energy generation cost over the 24-hour period, making it the most cost-effective method. POA has the third lowest cost, about 2.8% higher than HBA as shown in Table 5. GOA and GTO have very minor cost differences from HBA, within 0.1–0.7%. MRFO and SEA are slightly more expensive, approximately 1.2–1.3% higher than HBA. SSOA has significantly higher costs, around 7.4% more than HBA across the 24 h. In summary, HBA is the most affordable method, followed closely by GOA, GTO, POA, MRFO, and SHO, which have small cost differences from HBA. SSOA is the only method substantially more expensive than HBA over the full time period. HBA continues to demonstrate its exceptional performance, securing the status of the best-performing method with the highest reliability, as evidenced by its minimal value of 3.38 × 10^− 12^. This signifies HBA as the top choice within the given context. Conversely, GTO registers the maximum value of 3.96 × 10^− 6^, indicating its position as the worst-performing method with the lowest reliability in Case 3. This elevated value designates GTO as the least favorable algorithm, showcasing its comparatively poorer performance and lower reliability within the third scenario. MRFO and HBA led the performances among the algorithms. Specifically, HBA achieved the top efficiency score of 95.305%, while MRFO was a close second highest at 95.303%, only 0.002% lower than HBA. GOA had the lowest performance efficiency of 95.021% which was 0.282% below the leading efficiency achieved by HBA. The remaining algorithms, GTO, POA and SSOA, achieved results that were all within 0.05% of the top score shared by HBA and MRFO. Therefore, while MRFO and HBA demonstrated the strongest performances in Case 3, GOA reflected the weakest result that was nearly three tenths of a percent lower than the top performing algorithms in this scenario. HBA and MRFO tied for the fastest computation times of 569.8339 and 1023 s respectively. However, POA, GOA and SEA recorded significantly slower times of 1067.4, 1041.2 and 874.0521 s respectively, between 300 and 500 s slower than HBA and MRFO. SSOA was the third fastest at 654.0057 s, still over 80 s slower than the leading algorithms. Therefore, across all test cases, HBA consistently demonstrated the best performance as the fastest optimization algorithm. The detailed analysis of energy dynamics in the third scenario underscores the sophisticated functioning of the microgrid system. Figure 24 provides a comprehensive overview of power consumption and dump power occurrences throughout the day, showcasing the system’s responsiveness to the variable nature of RESs, particularly solar and wind. One notable aspect is the effective utilization of surplus power by the proposed EMS. As the generated power surpasses the immediate load demand and storage components reach full capacity, the EMS seamlessly redirects excess energy to auxiliary loads. The allocation of surplus power to the dummy load, as illustrated in the figures, serves a dual purpose—preventing overcharging, which could negatively impact the dummy load, and optimizing the overall energy utilization strategy. Figures 25, 26 delve into the SOC for both the BESS and SCs. At the start of the day, the supercapacitor’s SOC is at a robust 0.9, maintaining this level by day’s end. Throughout the day, the supercapacitor plays a crucial role in covering the load demand, with its lowest SOC observed at 1 pm, reaching 0.875 using the SHO algorithm. Notably, this period aligns with the peak load, demonstrating the supercapacitor’s active contribution during high-demand phases.

Conversely, the BESS starts the day with an SOC of 0.8 and concludes with the same value by day’s end, displaying a distinctive divergence in charging patterns compared to the supercapacitor. The BESS contributes significantly to covering the load demand during the day, with its lowest SOC recorded at 1 pm, reaching 0.58 using the HBA algorithm, aligning with the peak load period. This divergence in charging patterns between the supercapacitor and BESS underscores their complementary roles in the microgrid. While the supercapacitor excels in rapid-response scenarios and maintaining a consistent high SOC, the BESS showcases its ability to handle prolonged high-demand periods, contributing significantly to load coverage. These figures collectively provide a comprehensive view of the energy dynamics in the third scenario, encapsulating power generation, dummy load utilization, and the distinct charging behaviors exhibited by the energy storage systems. The EMS’s ability to efficiently manage surplus power and prevent overcharging contributes to the overall resilience and adaptability of the microgrid system in response to varying energy demands and storage capacities.

**Table 5:** Daily operational costs of seven algorithms in the 3rd scenario.

Time (hr)	Operating costs ($)						
	GOA	GTO	HBA	MRFO	POA	SHO	SSOA
1	0.88860	0.88860	0.88860	0.88860	0.88860	0.88860	0.88860
2	1.39322	1.39322	1.39322	1.39322	1.39322	1.39322	1.39322
3	1.94614	1.94614	1.94614	1.94614	1.94614	1.94614	1.94614
4	2.50445	2.50445	2.50445	2.50445	2.50445	2.50445	2.50445
5	3.11124	3.11124	3.11124	3.11124	3.11124	3.11124	3.11124
6	3.87117	3.87117	3.87117	3.87117	3.87117	3.87117	3.87117
7	4.88925	4.88925	4.88925	4.88925	4.88925	4.88925	4.88925
8	5.89769	5.89769	5.89769	5.89769	5.89769	5.89769	5.89769
9	6.99027	6.99027	6.99027	6.99034	6.99027	6.99265	6.99027
10	8.36319	8.36318	8.36319	8.36545	8.36319	8.36557	8.36318
11	10.10613	10.10678	10.10613	10.11263	10.10613	10.10851	10.10613
12	12.27750	12.27843	12.27750	12.29585	12.27750	12.27988	12.27750
13	18.83550	18.83655	18.83550	19.01222	19.80146	19.19492	21.15805
14	21.56461	21.57096	21.56460	21.74150	22.52820	21.92206	23.88450
15	23.89641	24.06935	23.89631	24.20714	24.86660	24.27438	26.41711
16	25.93708	26.18354	25.93851	26.27116	26.90553	26.31720	28.45106
17	27.66809	27.91480	27.66949	28.09244	28.63677	28.04850	30.17883
18	29.31511	29.55171	29.31272	29.76394	30.28143	29.69610	31.81591
19	30.46629	30.70795	30.46613	30.91408	31.42805	30.84618	32.96197
20	31.20125	31.44141	31.20104	31.64445	32.16330	31.58145	33.69728
21	31.87320	32.11103	31.87296	32.31119	32.83556	32.25377	34.36961
22	32.48497	32.72022	32.48468	32.91721	33.44767	32.86594	34.98179
23	33.04283	33.27530	33.04249	33.46888	34.00592	33.42423	35.54010
24	33.54698	33.77649	33.54659	33.96645	34.51046	33.92884	36.04472

**Fig. 27:**
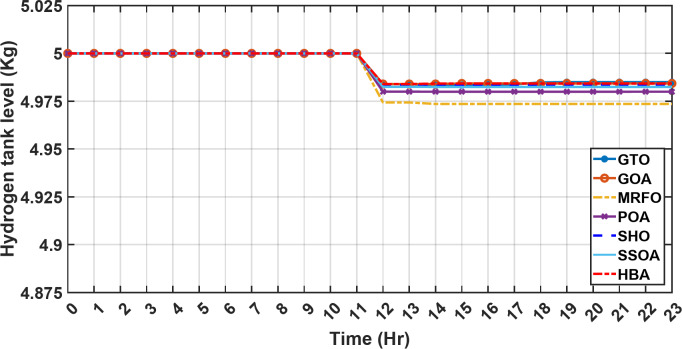
The fluctuations in the hydrogen tank level over the entire duration of a day in the third scenario.

**Fig. 28:**
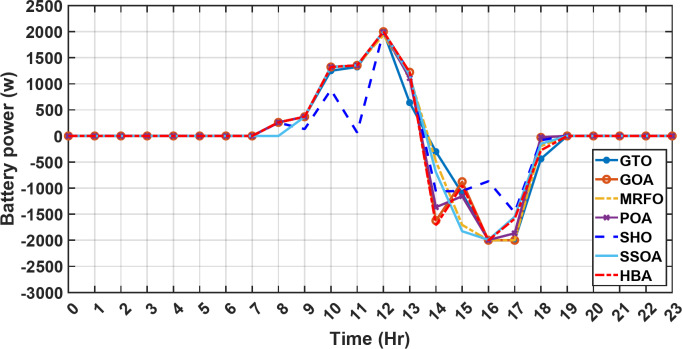
The fluctuations in the power of the BESS over the entire duration of a day in the third scenario.

**Fig. 29:**
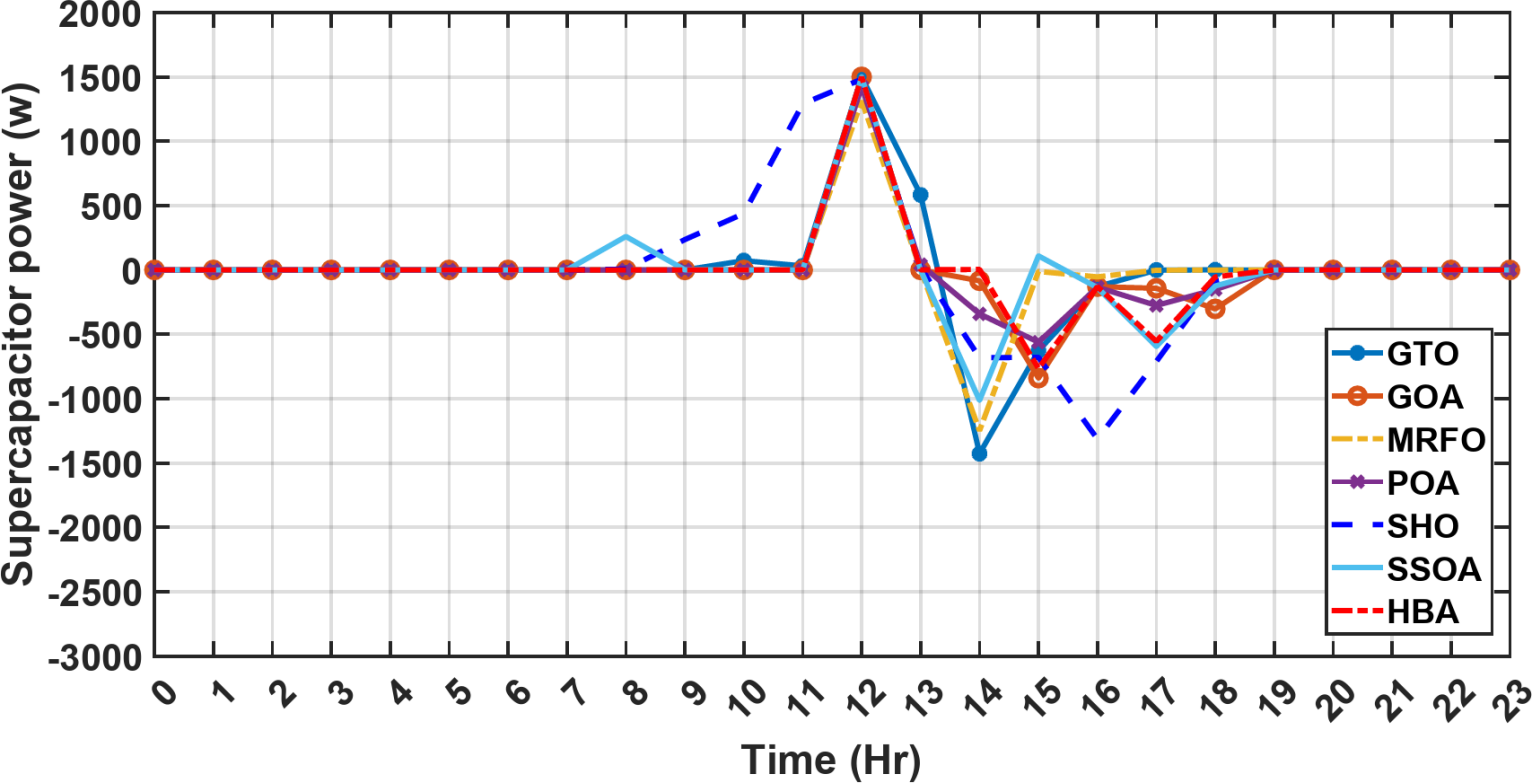
The fluctuations in the power of the supercapacitor over the entire duration of a day in the third scenario.

**Fig. 30:**
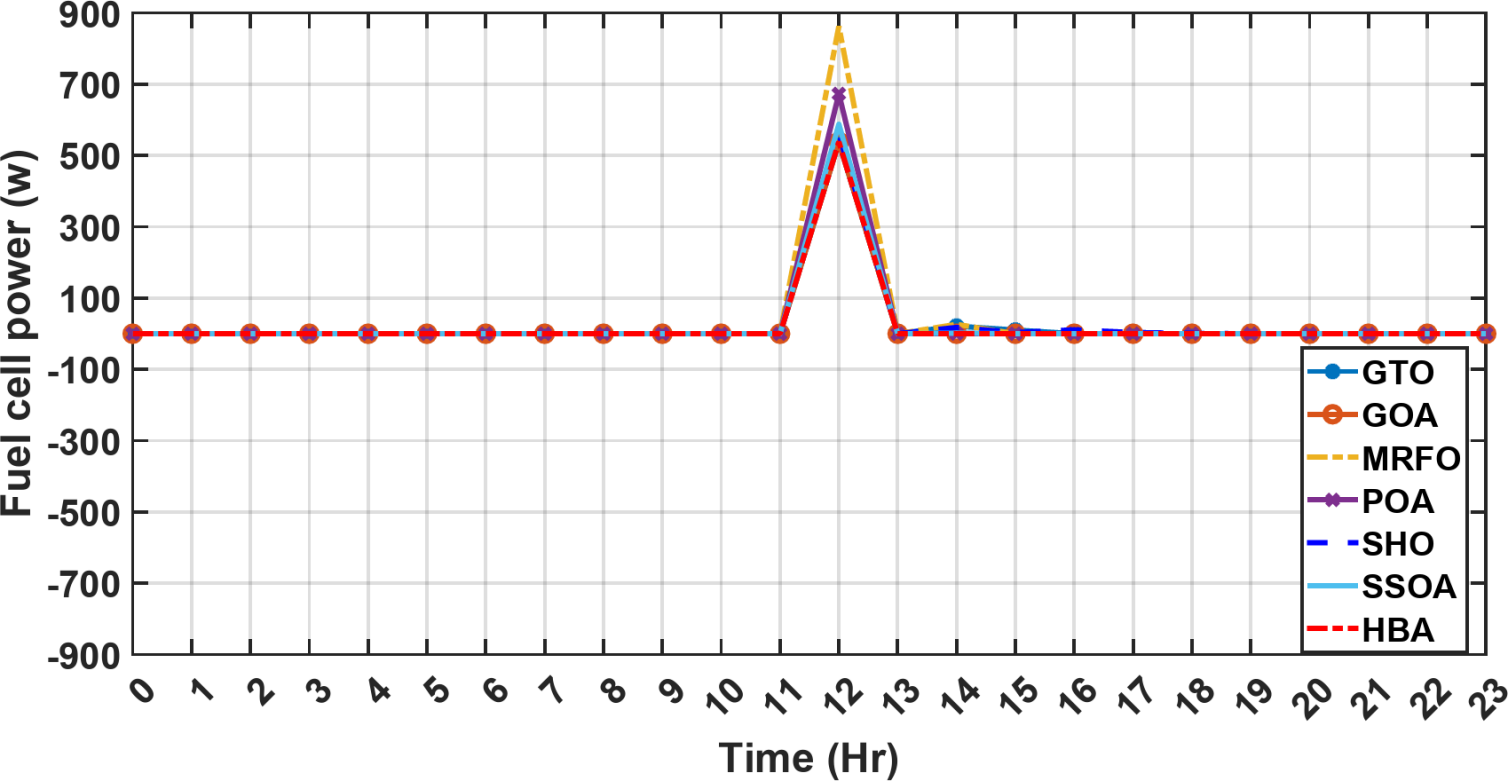
The fluctuations in the power of fuel cell over the entire duration of a day in the third scenario.

In Fig. 27, the hydrogen tank level is depicted, starting at 5 kg and gradually decreasing to 4.975 kg by the end of the day. This scenario underscores the inherent challenges associated with relying on backup sources, including both the BESS and SCs. The specific power contributions of the BESS are detailed in Fig. 28, while Fig. 29 provides a representation of the power contributed by the supercapacitor. It is observed that the BESS’s highest power contribution occurred at 1 pm, reaching 2000 W as shown in Fig. 28. Simultaneously, the highest power consumption during charging was recorded between 4 pm and 5 pm, also at 2000 W. This dynamic interaction showcases the BESS’s versatility in both supplying power during peak demand and efficiently managing its charging process. Similarly, Fig. 29 illustrates the supercapacitor’s power dynamics, with the highest contribution at 1 pm, reaching 1500 W. The highest power consumption during charging was observed at 2 pm, totaling 1500 W. These detailed power profiles highlight the nuanced behaviors of the supercapacitor in balancing energy contributions and withdrawals across varying demand periods. Figure 30 reveals a noteworthy observation for this specific scenario, the electrolyzer generated hydrogen at 12 pm, precisely during the peak load period. This indicates a strategic utilization of excess power to produce hydrogen, potentially for storage or utilization in other applications. The overall dynamics of these backup sources, including the strategic diversion of surplus power to the dummy load to prevent overcharging, emphasize their crucial role in ensuring the reliability and resilience of the energy system. The scenario effectively demonstrated the system’s capability to navigate situations where both reserve and primary sources are insufficient to meet energy demand or manage excess power. This underscores the significance of robust load management strategies and highlights the system’s adaptability in handling challenging conditions to maintain operational stability.

### Factors evaluating the EMS

The proposed EMS has demonstrated remarkable efficiency in optimizing operational costs while effectively balancing the delicate interplay between energy supply and demand. To rigorously evaluate its capabilities in handling dynamic scenarios, the system underwent rigorous testing across three distinct operational cases, each designed to assess its responsiveness to sudden changes and its efficacy in managing critical situations. One of the pivotal assessments involved subjecting the system to a state of severe powerlessness, deliberately challenging its load-shedding capabilities. This critical assessment aimed to gauge the system’s speed in making decisive load-shedding decisions during adverse conditions, which is crucial for maintaining grid stability. The results of this test showcased the system’s adeptness in swiftly shedding loads to mitigate the impact of power shortages. Another critical aspect was evaluating the system’s response to overflow situations, a key consideration to prevent overcharging and extend the lifespan of the energy storage system. The capability to manage overflow is vital for sustaining the health and performance of the energy storage infrastructure, avoiding premature degradation, and ensuring long-term reliability. The system not only effectively managed overflow scenarios but also succeeded in maintaining the LPSP within acceptable limits. LPSP serves as a vital reliability indicator, and Table 6 provides a comprehensive overview of LPSP values for the seven algorithms employed across the three operational conditions. Notably, the HBA emerged as the top performer among the algorithms, consistently achieving the best LPSP values across all operational cases. The standout performance of the HBA underscores its effectiveness in consistently meeting operational demands and maintaining reliability even in challenging circumstances. This exceptional performance is further corroborated by the efficiency metrics presented in Table 7, where HBA demonstrated the highest efficiency among the proposed algorithms in all operational scenarios. Moreover, the convergence time, a critical metric for evaluating algorithmic performance, was diligently considered. In the realm of algorithm performance evaluation, convergence time holds paramount importance. The speed at which an algorithm reaches a solution is pivotal in preserving network integrity, reducing outage durations, and enhancing overall operational conditions. The HBA outperforms other algorithms by achieving the lowest convergence time, clocking in at 569.3516 s during the first operational case as shown in Table 8.

In essence, the comprehensive testing and evaluation of the proposed EMS underscores its robustness and effectiveness in real-world operational scenarios. Its superior performance in load shedding, overflow management, and reliability indicators positions it as a promising solution for enhancing the resilience and efficiency of electrical networks. The remarkable efficiency demonstrated by the HBA further highlights its potential as a key component in advanced EMSs. Its ability to navigate challenging scenarios and deliver optimal results across various metrics positions it as a reliable and effective tool for ensuring the stability, efficiency, and resilience of energy systems in dynamic operational environments.

**Table 6:** LPSP values for the seven algorithms across three distinct operational cases.

Operational cases	LPSP						
	GOA	GTO	HBA	MRFO	POA	SEA	SSOA
Case 1	1.17e–8	2.70e–7	7.90e–10	1.33e–6	3.77e–7	6.72e–9	2.78e–6
Case 2	1.07e–08	3.81e–11	0	4.21e–11	5.74e–7	2.52e–6	9.56e–5
Case 3	1.20e–07	3.96e–06	3.38e–12	6.65e–06	2.06e–10	4.75e–05	2.22e–06

**Table 7:** Efficiency assessment of the seven algorithms across three unique operational scenarios.

Operational cases	Efficiency						
	GOA	GTO	HBA	MRFO	POA	SEA	SSOA
Case 1	0.95890	0.95827	0.95893	0.95826	0.95563	0.95891	0.95663
Case 2	0.96691	0.96650	0.96691	0.96689	0.96684	0.96656	0.96320
Case 3	0.95021	0.95150	0.95305	0.95303	0.95037	0.95007	0.95000

**Table 8:** Convergence time analysis for the seven algorithms across the three operational scenarios.

Operational cases	Convergence time (sec)						
	GOA (sec)	GTO	HBA	MRFO	POA	SEA	SSOA
Case 1	1058.4	2062.5	569.3516	998.6014	993.4645	835.0582	640.2090
Case 2	1024.5	2029.5	572.4453	1009.3	982.2214	917.0254	617.4670
Case 3	1041.2	2035 s	569.8339	1023	1067.4	874.0521	654.0057

## Conclusion

This paper implemented an advanced HRES, seamlessly integrating PV panels and WTs as primary power sources. To ensure a dependable and uninterrupted power supply, the system integrated three backup sources: batteries, HESSs, and SCs. Employing state-of-the-art optimization techniques, the system dynamically determined the most efficient energy flow during its operational phases. Seven distinctive algorithms are employed to identify the optimal solution, with a keen focus on two vital objective functions: minimizing operating costs and reducing the LPSP. Moreover, the study meticulously evaluated algorithm performance across nine benchmarks, with the ultimate goal of pinpointing the optimal algorithm for the hybrid renewable power system under scrutiny. The comprehensive evaluation of the proposed EMS spanned three distinct operational scenarios. In the initial scenario, power demand was met through a combination of primary and backup energy sources. In the second scenario, challenges arise as power demand surpasses generated capacity, attributed to charge limitations on all energy storage systems, rendering them unable to supply power to the load. The third scenario occurred when generated power exceeded the demand for immediate power, leading to the saturation of storage system components. Here, the proposed EMS redirects excess power to dummy loads, actively preventing overcharging and concurrently enhancing grid stability. Based on the analysis of the three test scenarios these points can be summarized:

- Through the implementation of optimization algorithms, the research achieves tangible cost savings by ensuring the efficient operation of the microgrid. The algorithms strategically determine when to activate backup systems, thus minimizing unnecessary energy expenditures. The economic gains go beyond theoretical models, providing practical insights into the potential financial benefits that can be realized through the judicious use of optimization algorithms in microgrid management.
- The system demonstrated highly effective overflow management, a critical facet for sustaining the health and performance of energy storage infrastructure. This sophisticated capability not only prevents premature degradation but also significantly contributes to the system’s long-term reliability. The ability to manage overflow scenarios ensures optimal functioning and extends the operational lifespan of the energy storage infrastructure.
- The system successfully maintained the LPSP within acceptable limits. LPSP, acting as a crucial reliability indicator, remained consistently within specified thresholds, reflecting the system’s ability to manage and sustain operational reliability.
- Under practical scenarios, the proposed study evaluates the HBA-based EMS as compared with other optimization techniques (GOA, GTO, MRFO, SSOA, SEA, POA) through a comparative analysis. This technical comparison involves considering the complexities of real-world energy systems, shedding light on the algorithmic efficiency and adaptability in addressing the intricacies of hybrid renewable energy microgrids.
- The HBA emerged as the top-performing algorithm, consistently achieving the best LPSP values across all operational cases. This underscores HBA’s effectiveness in meeting operational demands and maintaining reliability, particularly in challenging scenarios.
- HBA showcased the highest efficiency among the proposed algorithms in all operational scenarios. This high efficiency underscores HBA’s high performance and effectiveness in optimizing energy systems compared to alternative algorithms. The superior efficiency of HBA translates into optimized energy generation processes, minimizing wastage and maximizing resource utilization.
- HBA outperformed other algorithms in terms of convergence time, registering the lowest time at 569.3516 s during the initial operational case. This rapid convergence is indispensable for preserving network integrity, reducing outage durations, and enhancing overall operational conditions. The quick convergence time highlights HBA’s efficiency in swiftly reaching optimal solutions, contributing to the overall stability and reliability of the energy system.
- In case 1, HBA demonstrated its cost-effectiveness and efficiency by achieving the lowest overall energy generation cost, coupled with the highest efficiency of 95.893%. Outperforming other algorithms by substantial margins, with percentage differentials ranging from 0.002 to 6.925%, HBA showcased its effectiveness in optimization. Its computation time was notably faster, ranging from 70 to 488 s quicker than alternative algorithms. Moving to Case 2, HBA attained the absolute lowest cost and matched the top efficiency score of 96.691% with MRFO. Additionally, HBA delivered the fastest optimization, solving 400 to 450 s faster than other methods. In the third scenario, HBA consistently outperformed alternatives, producing the minimum average cost, maintaining the highest efficiency of 95.305%, and solving the optimization problem 300 to 500 s ahead of alternative algorithms. These results underscore HBA’s versatility and effectiveness, positioning it as a reliable choice for optimizing energy systems across various operational scenarios.

Nominal values of the EMS components.

**Table:** 

HEPMSG parameters			
P	Number of pairs of poles	6	
P_n_	Rated power	2500 W	
$\:{\omega\:}_{b}$	Base speed	2000 rpm	
R_a_	Stator winding resistance per phase	0.75 $\Omega$	
R_f_	Excitation winding resistance	2.82 $\Omega$	
L_d_	d-axis stator winding inductance	3.6 mH	
L_q_	q-axis stator winding inductance	5.1 mH	
L_f_	Excitation winding inductance	54 mH	
M_sf_	Mutual field – armature inductance	7 mH	
$\:{{\uplambda\:}}_{\text{P}\text{M}}$	Maximum value of the flux produced by the permanent magnet in a stator winding	100 mWb	
PV parameters			
N_s_	Number of series cells		100
N_p_	Number of parallel cells		12
R_s_	Pv series resistance		0.00152 $\Omega$
R_p_	Pv shunt resistance		100 $\Omega$
I_sc_	Short circuit current		4.8 A
A	Diode ideal factor		2
I_o_	Diode reverse saturation current		2.85 × 10^− 5^
Lead- acid battery parameters			
Nominal power			2000 W
Nominal voltage			200 V
Rated capacity			50 Ah
Nominal discharging current			10 A
Internal resistance			0.04 $\Omega$
Supercapacitors parameters			
Nominal power			1500 W
Rated capacitance			100 F
Equivalent DC series resistance			0.0089 $\Omega$
Rated voltage			60 V
Number of series capacitors			5
Number of parallel capacitors			5
Hydrogen system parameters			
Nominal power of fuel cell			1260 W
Nominal voltage of fue cell			24 V
Number of cells			42
Electrolyzer nominal power			3 Kw

## Data Availability

The data used to support the findings of this study are available from the corresponding author upon request.

## Abbreviations

RESs: Renewable energy systems
HRES: Hybrid renewable energy system
ESS: Energy storage system
FCs: Fuel cells
DERs: Distributed energy resources
WFSM: Wound field synchronous machine
SEA: Stochastic evolutionary algorithm
PSO: Particle swarm optimization
GTO: Giant trevally optimizer
ITLBO: Improved teaching and learning-based optimization
TLBO: Teaching and learning-based algorithm
ASSA: Adaptive salp swarm algorithm
ALO: Ant lion optimization
ABC: Artificial bee colony
RSA: Reptile search algorithm
LCOE: Levelized cost of energy
RESs: Renewable energy sources
SCs: Supercapacitors
O&M: Operating and maintenance
FLC: Fuzzy logic controller
IPMC: Intelligent power management control
COE: Cost of electricity
LOLP: Loss of load probability
FPA: Flower pollination algorithm
TNPV: Total net present value
PEMFC: Proton-exchange membrane fuel cell
BESS: Battery energy storage system
SESS: Supercapacitor energy storage system
HEPMSM: Hybrid excitation permanent magnet synchronous machine
PMSM: Permanent magnet synchronous machine
*N*_*it*_: Number of iterations
GOA: Gazelle optimization algorithm
HBA: Honey badger algorithm
MPPT: Maximum power point tracking
HTL: Hydrogen tank level
WECS: Wind energy conversion system
ANN: Artificial neural network
NPC: Net present cost
LCH: Levelized cost of hydrogen
BIPV: Building integrated photovoltaic
MSO: Modified snake optimization
EGS: Energy generation system
DR: Demand response
DSM: Demand side management
REMS: Residential energy management system
MRFO: Manta ray foraging optimization
POA: Pelican optimization algorithm
SHO: Sea horse optimizer
SSOA: Synergistic swarm optimization algorithm
AGTO: Artificial gorilla troops optimizer
ZOA: Zebra optimization algorithm
SOC: State-of-charge
ANFIS: Adaptive neuro-fuzzy inference system
FSIA: Flamingo swarm intelligence algorithm
WTs: Wind turbines
HESSs: Hydrogen energy storage systems
LPSP: Loss of power supply probability
DERs: Distributed energy resources
EMS: Energy management system
FCSEV: Fast charging station for electric vehicles
d-q axis: Direct-quadrature axis
*F*_*f*_: Fitness function
